# Full vision adaptation in mixed-light conditions enabled by dynamic water adsorption/desorption

**DOI:** 10.1038/s41467-026-73217-7

**Published:** 2026-06-09

**Authors:** Jia Zhu, Wantao Liu, Wanxin Huang, Xiangjie Chen, Xuewei Feng, Xin Luo, Kai Xu, Min Gao, Haifeng Ling, Chaoyun Song, Huanyu Cheng, Yuan Lin

**Affiliations:** 1 School of Materials and Energy, University of Electronic Science and Technology of China, Chengdu, China; 2 Yangtze Delta Region Institute (Quzhou), University of Electronics Science and Technology of China, Quzhou, China; 3 Energy and Information Materials Key Laboratory of Sichuan Province, University of Electronic Science and Technology of China, Chengdu, China; 4 School of Automation and Engineering, University of Electronic Science and Technology of China, Chengdu, China; 5 Nanjing University of Posts & Telecommunications (NJUPT), Nanjing, China; 6 School of Mechanical Engineering, Shanghai Jiao Tong University, Shanghai, China; 7 Guangdong Provincial Key Laboratory of Magnetoelectric Physics and Devices, School of Physics, Sun Yat-sen University, Guangzhou, China; 8 State Key Laboratory of Optoelectronic Materials and Technologies, School of Physics, Sun Yat-sen University, Guangzhou, China; 9 Department of Engineering, King’s College London, London, UK; 10 Department of Engineering Science and Mechanics, The Pennsylvania State University, University Park, Pennsylvania USA; 11 Medico-Engineering Cooperation on Applied Medicine Research Center, University of Electronics Science and Technology of China, Chengdu, China

**Keywords:** Electronic devices, Sensors and biosensors

## Abstract

Mimicking the human eye’s ability to autonomously adapt to diverse and mixed illumination conditions remains a fundamental challenge in artificial vision systems. Although substantial progress has been made in materials and device engineering, current adaptive vision architectures still depend heavily on complex circuitry or algorithms and are typically restricted to uniform illumination owing to the strong intensity-dependence of photosensitivity. Here, this work presents a highly adaptive TiO₂/PEDOT:PSS photomemristor that leverages the tunable conductivity of PEDOT:PSS together with the optoelectronic response of TiO₂. The photothermal effect dynamically modulates the water absorption/desorption equilibrium in PEDOT:PSS, enabling reversible suppression or enhancement of photosensitivity under bright or dim illumination, respectively. By combining with artificial neural networks (ANNs), the artificial vision system based on TiO₂/PEDOT:PSS photomemristor arrays achieves a high accuracy of 91.3% in image recognition under mixed-light conditions—without the need for complex circuitry or algorithms. This work may establish a new approach for designing autonomous, efficient, and high-performance neuromorphic vision systems to advance the development of autonomous driving and humanoid robots.

## Abstract (web summary)

Mimicking human eye’s ability to autonomously adapt to diverse and mixed illumination conditions remains a challenge in artificial vision systems. Here, the authors present an self-adaptive TiO₂/PEDOT:PSS photomemristor with vision adaptation to achieve accurate image recognition in a mixed-light environment.

## Introduction

Visual perception is one of the most important features of biological systems, including humans. Studies have revealed that over 80% of external environmental information is acquired through the visual system^1,2^. Designing artificial vision systems with similar features to biological systems is highly demanding for cutting-edge applications, including intelligent manufacturing, autonomous driving, and medical diagnostics. Earlier research on photodetectors has primarily focused on enhancing static performance metrics such as sensitivity and operational wavelengths, attempting to approach or even exceed those of human vision^3,4^. Owing to its superior sensitivity and dynamic adaptability, the human eye can process visual information under diverse illumination conditions, exhibiting a photodetection dynamic range exceeding 160 dB^5,6^. This capability allows humans to effectively respond to various illumination scenarios, such as watching a movie in a dark theater or driving in the glare of oncoming headlights^7^. However, conventional electronic photodetectors largely lack this fundamental capability of biological vision—the dynamic self-adaptation to complex and varying environments^8–10^. Until very recently, adaptation has been recognized as one of the key aspects in the design of artificial vision systems^11^. However, conventional photodetectors in previous literature reports have fixed photosensitivity and lack the capacity of adapting to different illumination conditions, resulting in image distortion and degraded recognition accuracy under fluctuating lighting conditions^12^.

Neuromorphic behavior has been widely recognized as a key route to achieving light or visual adaptation^13^. Tremendous progress has been made in the design and fabrication of neuromorphic devices, including two-terminal diodes or memristors and three-terminal field-effect transistors (FETs) or organic electrochemical transistors (OECTs)^14–18^, which primarily exploit the hysteresis behavior of semiconductors^14^. For instance, a MoS₂-based phototransistor has demonstrated tunable photosensitivity by introducing charge trap states to regulate the photocurrent. However, the adaptation of the MoS₂-based phototransistor requires external bias modulation, fundamentally restricting its feasibility in practical implementations. Even though adaptation in a P3HT/MAPbI₃ heterostructure has been achieved by leveraging the antagonistic interplay between a PN and Schottky junction without the need for external bias modulation, its performance remains highly sensitive to the thickness of P3HT with very low tolerance^16^. Furthermore, its limited tunable range of photosensitivity necessitates extra preprocessing and algorithms to achieve high recognition accuracy. Given these limitations, the development of neuromorphic vision devices with embodied adaptation to varying or even mixed illumination still remains a crucial challenge.

This work presents a highly adaptive TiO₂/PEDOT:PSS-based optoelectronic synapse, manifested by a rapid adaptation time and a significant suppression of photoresponse in bright light, to enable accurate vision recognition in complex illumination environments. This superior dynamic performance is enabled by the unique inorganic/organic heterojunction architecture. The TiO_2_/PEDOT:PSS interface provides an ideal platform for regulating the water adsorption/desorption dynamics on PEDOT:PSS, which in turn precisely modulates the resistance and postsynaptic current of the photomemristor. Specifically, under bright light, the device exhibits swift adaptation characterized by a rapidly decaying postsynaptic current, which even decreases below the baseline dark current. This embodied adaptation, quantified by its speed and efficiency, surpasses that of previously reported vision-adaptive synaptic devices. It enables the dynamic amplification of subtle low-intensity light signals while simultaneously suppressing high-intensity background. Such functionality effectively mitigates interference from brightness variations, markedly enhancing recognition accuracy in complex environments.

## Results

### Bioinspired visual adaptation

**Fig. 1:**
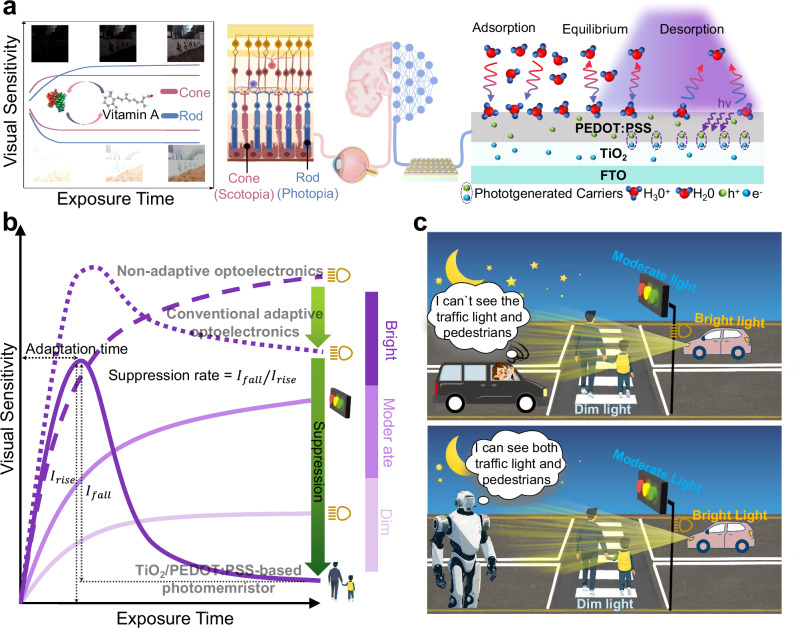
The bioinspired vision-adaptive optoelectronic synapse designed for image recognition in mixed-light environments. **a** Schematics showing the role of scotopia and photopia in the adaptation of human visual systems and the highly adaptive optoelectronic synapse based on the TiO_2_/PEDOT:PSS photomemristor. Inspired by rhodopsin, the visual adaptation of the proposed optoelectronic synapse is achieved via dynamic conductivity change in PEDOT:PSS through light intensity-controlled water adsorption and desorption. The schematic was created by BioRender.com. **b** Comparison of the photoelectricity characteristics between the TiO_2_/PEDOT:PSS photomemristor and other non-adaptive and adaptive optoelectronics reported previously in dim, bright, mixed-light environments. **c** Niche application of the image recognition provided by the vision-adaptive optoelectronic synapse in mixed-light environments. Part of the schematic in (**b**, **c**) was generated using CleanPNG.com.

The extraordinary photopic–scotopic adaptability of the human visual system relies on the unique regenerative mechanisms of photopigments in rod and cone cells. Under bright illumination, rhodopsin in rod cells undergoes extensive bleaching followed by slow regeneration^19,20^, leading to temporarily suppressed photosensitivity (Fig. 1a). Meanwhile, photopigments in cone cells experience similar photochemical reactions but regenerate much faster than rhodopsin, enabling the rapid recovery to maintain high-acuity color vision and fine-detail perception in bright conditions^21^. In dim light, the regeneration rate of rhodopsin exceeds its bleaching rate, with substantially increased concentration in the retina, leading to the restoration of photosensitivity of rod cells to realize scotopic adaptation. Overall, the dynamic bleaching/regeneration of photopigments and the complementary coordination between rod and cone cells endow the human eye with remarkable adaptability across a broad range of illumination intensities.

Inspired by the human eye, tunable photosensitivity has been explored to develop adaptive optoelectronic devices and artificial visual systems that can enhance or suppress photosensitivity under dim or bright illumination, respectively^14,16,22^. Different from conventional optoelectronic devices, adaptive devices exhibit saturated photocurrent responses and suppressed photosensitivity with increasing illumination intensity (Fig. 1b). However, despite this suppression, the photocurrent of previously reported adaptive devices under bright light remains substantially higher than that under dim illumination. Consequently, these devices are incapable of detecting low or moderate-brightness objects in the background of strong light. In other words, their adaptation is effective only under fixed and uniform lighting conditions. This limitation severely restricts the applicability of conventional adaptive optoelectronic devices in complex real-world environments, such as recognizing pedestrians (in low brightness) and traffic lights (in moderate brightness) amid oncoming high beams (in high brightness) (Fig. 1c).

Inspired by the photopigment bleaching and regeneration mechanism of the human eye, this work introduces a TiO₂/PEDOT:PSS heterojunction photomemristor to achieve fully self-adaptive behavior through a dynamic water adsorption/desorption process. In this design, the equilibrium between water adsorption and desorption on the PEDOT:PSS surface is modulated by light intensity, dynamically tuning photosensitivity via the concentration of hydronium ions. Under dim illumination, water adsorption dominates, corresponding to a high concentration of hydronium ions and high conductivity and photosensitivity. In comparison, water desorption starts to dominate over adsorption under bright illumination, reducing the hydronium ion concentration for suppressed conductivity and photosensitivity. Remarkably, this bioinspired self-regulation mechanism results in unprecedented vision adaptation, manifested by significantly suppressed photocurrent and photosensitivity in bright light that are even lower than those in the dim light. Such a unique capability to amplify weak or moderate optical signals while suppressing strong light effectively mitigates interference from bright backgrounds. Consequently, the proposed photomemristor not only mimics but surpasses the human visual system by emulating the photopigment bleaching and regeneration process. Specifically, dynamic modulation of the conductivity in the TiO₂/PEDOT:PSS-based photomemristor was achieved through the reversible absorption and desorption of water molecules in the PEDOT:PSS layer induced by the photothermal effect. As a result, the TiO₂/PEDOT:PSS-based photomemristor exhibits record-breaking suppression of photocurrent under bright illumination and demonstrates robust photopic–scotopic adaptation, significantly improving recognition accuracy under complex lighting conditions. To quantitatively and comprehensively highlight the bioinspired design advantages and performance of the TiO₂/PEDOT:PSS photomemristor, a detailed comparison between the as-fabricated device and the human visual system, in terms of structural and functional features, is provided in Table S1. The results show that the TiO₂/PEDOT:PSS-based photomemristor exhibits significantly shorter adaptation times in both dark and bright environments than the human visual system, while maintaining comparably low power consumption. In addition, the TiO₂/PEDOT:PSS-based photomemristor also features a simple device structure and is compatible with scalable fabrication, which is advantageous for practical applications.

### Characterization of the TiO_2_/PEDOT:PSS-based photomemristor

**Fig. 2:**
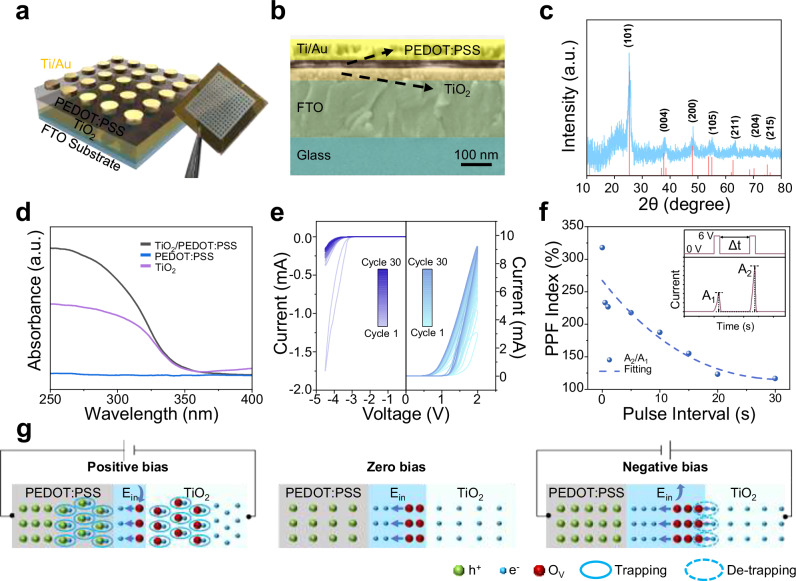
Material and electrical characterizations of the TiO₂/PEDOT:PSS-based photomemristor. **a** Schematic illustrating the structure of the TiO₂/PEDOT:PSS-based photomemristor array and the corresponding optical image shown in the inset. **b** Cross-sectional SEM image of the multilayered FTO/TiO_2_/PEDOT:PSS/Ti/Au structure in the TiO₂/PEDOT:PSS-based photomemristor. **c** GIXRD patterns of the anatase TiO_2_ layer. **d** UV–vis absorption spectra of the TiO_2_, PEDOT:PSS layer, and TiO_2_/PEDOT:PSS heterojunction. **e** *I* − *V* hysteresis loops of the TiO_2_/PEDOT:PSS-based photomemristor synapse under 30 consecutive negative (left, from 0 to −4.5 V) and positive (right, from 0 to 2 V) voltage sweeps. **f** PPF index of the TiO₂/PEDOT:PSS-based photomemristor as a function of the pulse interval (*Δt*). PPF is defined as the peak current ratio between the first and second pulse. The pulse amplitude (*V*_bias_) is set to 6 V. **g** Schematics illustrating the origin of the synaptic behavior in the TiO₂/PEDOT:PSS-based photomemristor under positive (left) and negative (right) bias voltages.

The adaptive optoelectronic synapse features a two-terminal memristor with a TiO₂/PEDOT:PSS heterojunction as the functional layer (Fig. 2a). The commercially available fluorine-doped tin oxide (FTO) glass serves as the bottom electrode, whereas the top Ti/Au electrode is fabricated by magnetron sputtering. The TiO₂ layer is prepared using a sol–gel process, followed by spin-coating and thermal annealing at 450 °C, as in the recent literature report^23^. The PEDOT:PSS layer is deposited by spin-coating and subsequent annealing at 130 °C under vacuum^24^. Detailed fabrication procedures are provided in the *Method* and Fig. S1. Cross-sectional SEM image reveals the multilayered structure of the photomemristor, with a thickness of 76 nm and 39 nm for the TiO₂ and PEDOT:PSS layers, respectively (Fig. 2b). Grazing-incidence X-ray diffraction (GIXRD) confirms that the TiO_2_ layer adopts the anatase phase (Fig. 2c), which can be attributed to the relatively low annealing temperature. Raman spectroscopy further confirms the anatase crystal structure of the TiO₂ film, with characteristic peaks located at 143, 399, 492, and 633 cm^−^¹ (Fig. S2), corresponding well to the typical vibrational modes of anatase TiO₂ (*E*_g_, *B*_1g_, *A*_1g_, and *E*_g_, respectively). Compared with the thermodynamically stable rutile phase, anatase TiO₂ possesses a smaller effective electron mass and higher electron mobility, both of which are favorable for rapid synaptic response^25^. In addition, anatase TiO₂ exhibits high surface reactivity due to abundant surface defects and oxygen vacancies, facilitating the formation of a high-quality heterojunction interface with the PEDOT:PSS layer^26,27^. Atomic force microscopy (AFM) measurements reveal that the TiO₂/PEDOT:PSS bilayer exhibits a smoother surface (root-mean-square roughness of 2.09 nm) than either the TiO₂ (≈3.05 nm) or PEDOT:PSS (≈4.41 nm) monolayers, confirming the superior interfacial quality of the heterojunction (Fig. S3). Overall, both SEM and AFM analyses demonstrate the exceptional flatness of the TiO₂ layer and its excellent interfacial compatibility with PEDOT:PSS—key to achieving high uniformity and reproducibility^28^. Ultraviolet–visible (UV–vis) absorption spectra (Fig. 2d) show significantly higher absorption in the UV region for the TiO₂/PEDOT:PSS heterojunction compared with the individual TiO₂ or PEDOT:PSS layers, indicating enhanced photoelectric sensitivity^29^. The combination of high electron mobility of TiO₂ and improved light absorption facilitates the optoelectronic synaptic behavior of the TiO₂/PEDOT:PSS-based photomemristor.

In biological neural systems, signal transmission along neurons—particularly through the axon—occurs via electrical impulses mediated by Na⁺ influx and K⁺ efflux across the cell membrane. When an action potential reaches the axon terminal (i.e., the synapse), it triggers the release of neurotransmitters from synaptic vesicles into the synaptic cleft through membrane fusion^30^. Upon receiving these neurotransmitters, the postsynaptic membrane undergoes potential modulation, generating either an excitatory or inhibitory postsynaptic potential (EPSP or IPSP), which in turn produces an excitatory or inhibitory postsynaptic current (EPSC or IPSC), respectively. The amplitude of EPSC or IPSC depends on the synaptic connection strength—known as the synaptic weight—which can be dynamically modulated by neuronal activity. This modulation, referred to as synaptic plasticity, underlies the processes of learning and memory. By analogy, the two terminals of the photomemristor (Au and FTO electrodes) act as the presynaptic and postsynaptic membranes, respectively, while its conductance corresponds to the synaptic weight. The conductance can be modulated by electrical pulses, mimicking presynaptic neuronal activity. The synaptic plasticity of the TiO₂/PEDOT:PSS-based photomemristor is evaluated through consecutive *I–V* sweeps (Fig. 2e). The TiO₂/PEDOT:PSS heterojunction exhibits clear hysteresis in *I–V* sweeps, indicating memristive behaviors. Moreover, the conductance decreases (or increases) progressively under repeated negative (or positive) voltage sweeps. These memristive characteristics can be explained by carrier migration and trapping at the TiO₂/PEDOT:PSS interface. This gradual and controllable conductance modulation is a key characteristic of oxygen-vacancy migration-based memristors, distinguishing our device from filamentary memristors and contributing to low device-to-device variability and extraordinary cycling endurance^31^. To further validate the oxygen-vacancy-based switching mechanism in our device, additional area-dependent electrical measurements are performed on devices with different electrode sizes. As shown in Fig. S4, the current of the TiO₂/PEDOT:PSS photomemristor increases with electrode area, indicating that conduction scales proportionally with device area. This result strongly suggests that conduction is dominated by a bulk transport process rather than by localized filamentary conduction, consistent with the proposed oxygen-vacancy migration mechanism in the TiO₂/PEDOT:PSS photomemristor. The ultraviolet photoelectron spectroscopy (UPS) results reveal the conduction band (C_B_) of TiO₂ (~3.74 eV) and the highest occupied molecular orbital (HOMO) of PEDOT:PSS ( ~ 3.16 eV) relative to the vacuum level (Fig. S5ac). The band gaps of TiO₂ and PEDOT:PSS are determined as 3.32 and 1.52 eV from UV–vis absorption spectra using Tauc plot analysis, assuming an indirect transition for TiO₂ and a direct transition for PEDOT:PSS (Fig. S5bd). Based on these experimentally derived parameters, the energy band alignment of the TiO₂/PEDOT:PSS heterojunction is constructed (Fig. S6), and the built-in electric field arising from carrier diffusion across the interface is schematically illustrated in Fig. 2g. Under positive bias, electrons are injected from the FTO electrode and trapped by oxygen vacancies of TiO_2_, which weakens the built-in electric field in the depletion region. Simultaneously, negative ions (PSS⁻) migrate directionally within the PEDOT:PSS layer and holes are efficiently injected from the Au electrode to the HOMO level of PEDOT:PSS to accumulate, resulting in the rise of the doping level and thus conductivity of PEDOT:PSS. These processes lead to a continuously increased conductance of the PEDOT:PSS-based memristor and its transition to the low-resistance state (LRS). Under negative bias, the heterojunction is reset to the high-resistance state (HRS) in a reverse process. The reversed field extracts the electrons that are trapped in oxygen vacancies, leading to the strengthening and widening of the built-in electric field. Concurrently, the negative bias also leads to the de-doping of PEDOT:PSS and consequently decreases conductivity. These processes build up the energy band barrier of the heterojunction, leading to decreasing conductivity until the heterojunction returns to the HRS.

Repeated stimulation can strengthen or weaken the synaptic weight, corresponding to synaptic excitation or depression. Depending on the pulse interval, synaptic plasticity can be categorized as short-term plasticity (STP) or long-term plasticity (LTP). To characterize the STP and LTP behaviors of the TiO₂/PEDOT:PSS-based memristor, two consecutive voltage pulses with a magnitude (i.e., *V*_bias_) of 6 V and a width (i.e., *P*_w_) of 100 ms are applied at varying intervals (*Δt*). The second postsynaptic current (PSC) is observed to be much higher than the first one, corresponding to the paired-pulse facilitation (PPF) phenomenon (Fig. 2f). The PPF effect weakens with increasing pulse interval. To quantitatively analyze this trend, the PPF index is defined as

1 $${PPFindex}={A}_{2}/{A}_{1}\times 100\%$$

where *A*_1_ and *A*_2_ denote the PSC amplitudes under the first and second pulses, respectively. A PPF index greater than 1 indicates enhanced response, which is beneficial for learning processes. The PPF index of the TiO₂/PEDOT:PSS memristor decreases markedly from 3.18 to 1.17 as the pulse interval increases from 10 ms to 30 s. Remarkably, the device maintains a PPF index of higher than 1 even at a 30-second interval, demonstrating exceptional synaptic plasticity.

**Fig. 3:**
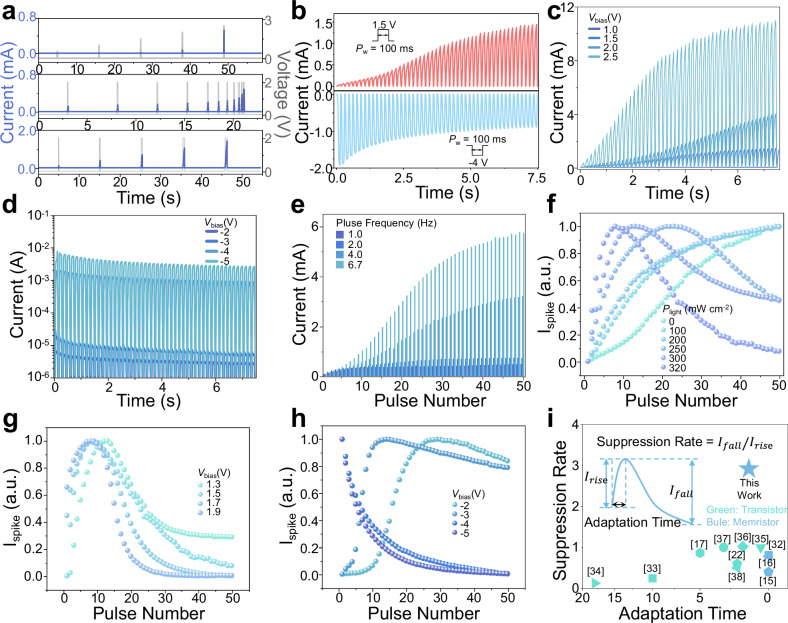
Synaptic and vision-adaptive characteristics of the TiO₂/PEDOT:PSS-based photomemristor. **a** Plasticity of the TiO₂/PEDOT:PSS-based photomemristor as a function of time, modulated by different sets of the pulse parameters (amplitudes (*V*_bias_), durations (*P*_w_), and interval (*Δt*)). **b** Excitatory postsynaptic current (EPSC) of the TiO₂/PEDOT:PSS-based photomemristor upon 50 positive (Top red, *V*_bias_ = 1.5 V) or negative (bottom blue, *V*_bias_ = −4 V) stimulation pulses (*P*_w_ = 100 ms, *Δt* = 50 ms). PSC response of the TiO₂/PEDOT:PSS-based photomemristor as a function of the time, modulated by 50 (**c**) positive (*V*_bias_ = 1, 1.5, 2, 2.5 V) and (**d**) negative (*V*_bias_ = −2, −3, −4, −5 V) voltage pulses with different amplitudes. **e** EPSC of the TiO₂/PEDOT:PSS-based photomemristor as a function of the voltage pulse number with different pulse frequencies: 1.0, 2.0, 4.0, and 6.7 Hz. **f** Normalized spike current response (*I*_spike_) of the TiO₂/PEDOT:PSS-based photomemristor in different light intensities: 0, 100, 200, 250, 300, and 320 mW cm^−2^ (*V*_bias_ = 1.5 V, *P*_w_ = 100 ms). Influence of (**g**) positive and (**h**) negative bias voltage on the normalized *I*_spike_ of the TiO₂/PEDOT:PSS-based photomemristor as a function of the pulse number upon light illumination of 320 mW cm^−2^. **i** Comparison of the suppression rate and adaptation time between the TiO₂/PEDOT:PSS-based photomemristor in this work and others in previous literature.

Neuronal synaptic plasticity depends on the strength of stimuli, modulated by the frequency, amplitude, and duration of presynaptic spikes. As a result, the synaptic plasticity of the TiO₂/PEDOT:PSS-based memristor is investigated under varying voltage pulse intervals, amplitudes, and widths (Fig. 3a). The PSC increases significantly with the increasing pulse amplitude, frequency, and width. For instance, at a fixed pulse interval of 10.9 s and width of 100 ms, increasing the pulse amplitude from 0.5 V to 2.5 V enhances the PSC from 593 nA to 5.46 mA (Fig. 3a, top). With a pulse amplitude and width of 2 V and 100 ms, reducing the pulse interval from 5 s to 100 ms results in an increase of PSC from 153 μA to 224 μA (Fig. 3a, mid). With a fixed pulse amplitude and interval of 2 V and 10 s, increasing the pulse width from 100 ms to 500 ms enhances the PSC from 102 μA to 1.42 mA (Fig. 3a, bot). Continuous stimulation experiments further elucidate synaptic behavior (Fig. 3b–e). Under successive positive (or negative) voltage pulses (*V*_bias_ = 1.5 or −4 V, *Δt* = 50 ms), the PSC progressively increases (or decreases) from 35 μA with the pulse number until saturation of 1.44 mA, corresponding to excitation (or depression) (Fig. 3b). This behavior resembles the biological transition from sensitization to habituation with sustained stimulation. The synaptic plasticity of the TiO₂/PEDOT:PSS memristor can be further modulated by continuous-pulse amplitude. The EPSC (or IPSC) responses under 50 consecutive positive (or negative) pulses of different amplitudes of 1/1.5/2/2.5 V (or −2/3/4/5 V) are further recorded (Fig. 3c, d). As the positive pulse amplitude increases from 1 V to 2.5 V, the time to reach saturated EPSC decreases from 5.40 s to 3.15 s, while the saturated EPSC increases from 1.21 mA to 8.23 mA (Fig. 3c). Similarly, larger negative pulses induce more pronounced IPSC decay (Fig. 3d), indicating stronger synaptic depression. Varying the pulse frequency produces an analogous modulation of synaptic weight: as the frequency increases from 1 Hz to 6.67 Hz, the EPSC at the 50th pulse increases from 0.48 mA to 5.74 mA (Fig. 3e). Collectively, these results confirm the robust synaptic plasticity of the TiO₂/PEDOT:PSS-based memristor and its extraordinary tunability through voltage pulse parameters.

### Visual adaptation of the TiO₂/PEDOT:PSS-based photomemristor

The optoelectronic properties of TiO₂ endow the TiO₂/PEDOT:PSS-based memristor with optoelectronic synaptic behavior, forming a photomemristor. Meanwhile, the micromorphology and conductivity of PEDOT:PSS depend on the water absorption, which can be further modulated by the photothermal effect. The combination of synaptic plasticity and tunable conductivity provides the TiO₂/PEDOT:PSS-based photomemristor with self-adaptation. The synaptic plasticity of the TiO₂/PEDOT:PSS-based photomemristor under various UV light intensities (i.e., *P*_light_) is examined (Figs. 3f and S7). Under identical voltage pulses (*V*_bias_ = 1.5 V, *P*_w_ = 100 ms, *Δt* = 50 ms), the postsynaptic current (PSC) of the TiO₂/PEDOT:PSS-based photomemristor increases with the number of pulses before saturation, when the light intensity is below 200 mW cm^−^². Remarkably, at higher illumination intensities, the PSC exhibits a distinct knee point (or maximum) before reaching a stable level as the pulse number increases. Moreover, the knee point shifts from the 23rd pulse to the 8th pulse as the light intensity increases from 250 to 320 mW cm^−^². The significantly suppressed synaptic plasticity under stronger illumination endows the TiO₂/PEDOT:PSS-based photomemristor with intrinsic light adaptation. Notably, the steady-state synaptic current under a light intensity of 320 mW cm^−^² (0.95 mA, in Fig. S7h) is substantially lower than the dark current (1.44 mA, in Fig. S7a), demonstrating the unprecedentedly suppressed synaptic plasticity and adaptation behavior of the device.

The key mechanism underlying light adaptation in the TiO₂/PEDOT:PSS-based photomemristor is the dynamic water absorption and desorption on PEDOT:PSS driven by the photothermal effect. With the increasing illumination intensity, water desorption is enhanced due to elevated temperature. The dehydration of PEDOT:PSS results in reduced conductivity and consequently a suppressed synaptic current (see detailed discussion in the following section). The light adaptation of the TiO₂/PEDOT:PSS-based photomemristor can also be modulated by electrical stimuli. At a fixed light intensity of 320 mW cm^−^², the suppression of synaptic enhancement, manifested by the ratio of the peak to the steady baseline, increases as the applied positive voltage rises from 1.3 V to 1.9 V (Figs. 3g and S8). The corresponding knee point of the PSC also shifts from the 13th to the 8th pulse.

Interestingly, under a fixed light intensity of 320 mW cm^−^², the PSC of the TiO₂/PEDOT:PSS-based photomemristor initially increases with the first few pulses when weak negative voltage pulses (−2 and −3 V) are applied (Figs. 3h and S9). This can be attributed to the large number of photogenerated carriers induced by UV illumination. As the number of pulses increases, the PSC saturates and subsequently decreases, revealing clear light adaptation. Under stronger negative voltage pulses (−4 and –5 V), the knee point in the PSC curve disappears, and the PSC is markedly suppressed under illumination due to enhanced adaptive behavior, which can be attributed to the strong inhibition induced by the stronger negative voltage.

To quantitatively evaluate the light-adaptive behavior of the photomemristor, a photosensitivity score^22^, *S*(n), is defined as:

2 $$S({{\mbox{n}}})=({I}_{{\mbox{n}}}^{{\mbox{photo}}}-{I}_{{\mbox{n}}}^{{\mbox{dark}}})/{I}_{{\mbox{n}}}^{{\mbox{dark}}}\times 100\%$$

where ${I}_{{{\rm{n}}}}^{{\mbox{photo}}}$ and ${I}_{{{\rm{n}}}}^{{\mbox{dark}}}$ represent the PSC under the *n*_th_ voltage pulse (1.5 V) with and without light illumination, respectively, and *n* denotes the pulse number. Overall, *S*(n) decreases with the increasing pulse number, regardless of light intensity (Fig. S10). However, larger *S*(n) values are observed under stronger illumination, indicating enhanced light adaptation. Notably, as the pulse number (i.e., adaptation time) increases, *S*(n) under high-intensity illumination (320 mW cm^−^²) rapidly decreases from 2527 before saturating at a negative value of −33.7, which is even lower than the saturation under weak illumination (e.g., 37.7 for 100 mW cm^−^²). The exceptionally suppressed PSC under strong illumination highlights the unprecedented light adaptation capability of the TiO₂/PEDOT:PSS-based photomemristor. Comprehensive comparison of the adaptation time and suppression rate (defined by the ratio of current fall and rise, *I*_*fall*_/*I*_*rise*_) between the TiO₂/PEDOT:PSS-based photomemristor and others in previous literature reports was made in Fig. 3i^32–38^. Collectively, these electrical, spectroscopic, and surface-chemical analyses consistently validate that the adaptive behavior of the TiO₂/PEDOT:PSS-based photomemristor arises from a light-regulated hydration–dehydration mechanism rather than morphological or purely thermal effects. By dynamically modulating the concentration of water-derived ionic species and thus the conductivity of PEDOT:PSS, the device achieves a self-regulating photosensitivity that underpins its robust and reversible visual-adaptation functionality.

### Working mechanism of the visual-adaptation in the TiO₂/PEDOT:PSS-based photomemristor

**Fig. 4:**
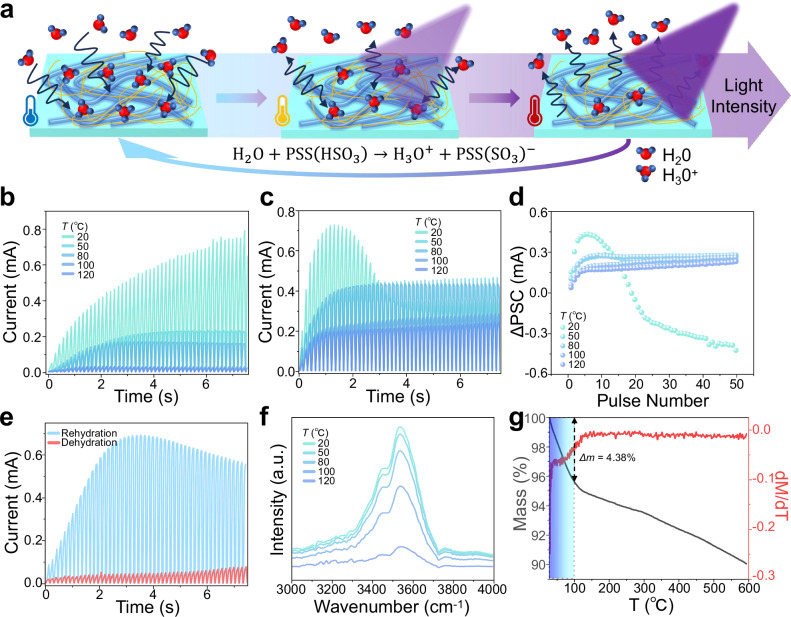
Vision-adaptation mechanism of the light intensity-regulated water absorption/desorption on PEDOT:PSS. **a** Schematic illustrating the photopic and scotopic adaptation of the TiO₂/PEDOT:PSS-based photomemristor via the light intensity-regulated water absorption/desorption on PEDOT:PSS. EPSC of the TiO₂/PEDOT:PSS-based photomemristor at different temperatures: 20, 50, 80, 100, and 120 °C (**b**) with or (**c**) without light illumination of 320 mW cm^−2^ (*V*_bias_ = 1.5 V, *P*_w_ = 100 ms, *Δt* = 50 ms). **d** Photocurrent (calculated by the difference between the EPSC with and without light illumination of 320 mW cm^−2^) of the TiO₂/PEDOT:PSS-based photomemristor as a function of the pulse number at different temperatures: 20, 50, 80, 100, and 120 °C. **e** EPSC of the TiO₂/PEDOT:PSS-based photomemristor before (red) and after (blue) rehydration. **f** In situ IR spectra and (**g**) thermogravimetric analysis (TA) of the TiO₂/PEDOT:PSS heterostructure at different temperatures.

The unprecedented light adaptation of the TiO₂/PEDOT:PSS-based optoelectronic synapse can be attributed to a self-regulated water absorption/desorption process that is coupled with the photoelectric conversion process. Under light illumination, photons are primarily absorbed by the TiO₂ layer, generating electron–hole pairs. The photogenerated carriers undergo efficient separation due to the strong built-in electric field at the heterojunction. Electrons are transported through the TiO₂ layer and collected by the FTO electrode, while holes are injected into the HOMO level of PEDOT:PSS and subsequently collected by the Au electrode, which results in a measurable photocurrent. These photogenerated carriers and photocurrent are also regulated by the applied voltage pulses, which are superimposed on the postsynaptic current (PSC) to result in the optoelectronic synaptic behavior^29^. In parallel with photoelectric conversion, the tunable conductivity of PEDOT:PSS by water absorption endows the TiO₂/PEDOT:PSS-based optoelectronic synapse with exceptional light adaptation^39–44^. Water absorption/desorption is a dynamic process on PEDOT:PSS, and its equilibrium relies on the temperature. With increasing light intensity, water desorption on the PEDOT:PSS film dominates over absorption due to the photothermal effect. This process reduces the hydronium ion (H₃O⁺) concentration and consequently leads to the decreased conductivity of PEDOT:PSS, which suppresses the synaptic current of the TiO₂/PEDOT:PSS-based photomemristor in strong light (Fig. 4a). Systematic infrared (IR) thermography measurements at four illumination intensities (0, 100, 200, and 320 mW cm^−^²) at a constant room temperature of 20 °C quantitatively evaluate the photothermal effect under illumination (Supplementary Movie 1 and Fig. S11). The results show that the temperature of the TiO₂/PEDOT:PSS photomemristor increases monotonically with illumination intensities. For example, after 7.5 s of illumination, the temperature of the photomemristor increases from 24.2 to 32.7 °C as the illumination intensity rises from 100 to 320 mW cm^-2^. Furthermore, its ultimate temperature also rises with increasing illumination duration. Collectively, these results confirm the presence of a pronounced photothermal effect in the TiO₂/PEDOT:PSS photomemristor, which can be attributed to the unique light absorption properties of PEDOT:PSS and TiO_2_. To verify this mechanism, the conductivity of the PEDOT:PSS film at different temperatures is examined (Fig. S12). It is found that the conductivity of PEDOT:PSS decreases with increasing annealing temperature. Furthermore, the PSC of the TiO₂/PEDOT:PSS-based memristor is measured at different temperatures (Fig. 4b). Under a voltage pulse of 1.5 V, the PSC in dark environments initially increases and then saturates with the pulse number, regardless of the temperature. However, the saturated PSC decreases significantly with increasing temperature, confirming the influence of temperature on the synaptic behavior of TiO₂/PEDOT:PSS-based memristors. Under UV illumination of 320 mW cm^−^², the PSC of the TiO₂/PEDOT:PSS-based memristor increases compared to the case without light for all temperatures except 20 °C, which can be attributed to the photocurrent (Fig. 4c). At 20 °C, the PSC of the TiO₂/PEDOT:PSS-based memristor rises and then drops dramatically with the pulse number. The PEDOT:PSS film is initially hydrated at 20 °C, so UV illumination disrupts the equilibrium between water absorption and desorption via the photothermal effect. This leads to a gradual dehydration of PEDOT:PSS and consequently decreased conductivity. When the influence from conductivity reduction outweighs the photocurrent, the photomemristor exhibits suppressed PSC and light adaptation. The similar change of PSC upon UV illumination (i.e., ΔPSC) at higher temperatures (i.e., 50, 80, 100, and 120 °C) confirms the contribution from the photocurrent and the variations in the photocurrent of TiO_2_/PEDOT:PSS-based photomemristor caused by temperature changes alone are negligible(Fig. 4d). Changing the temperature back to 20 °C does not result in the retrieval of PSC suppression and light adaptation for the dehydrated TiO₂/PEDOT:PSS-based photomemristor. However, immersing the TiO₂/PEDOT:PSS-based photomemristor in highly humid environments recovers its light adaptation (Fig. 4e). These results indicate that the light adaptation originates from the temperature-regulated water absorption rather than the temperature itself. Further reliability evaluation of the TiO₂/PEDOT:PSS-based photomemristor under varying environmental conditions measures its photoresponse at three relative humidity (RH) levels (30%, 54%, and 92%) under UV illumination (320 mW cm^−^²) (Fig. S12). Notably, the adaptive knee point shifts from the 6th pulse (0.9 s) at 30% RH to the 11th pulse (1.65 s) at 92% RH, indicating a clear humidity-dependent delay. The adaptive behavior occurs earlier in low-humidity environments due to the limited ambient water molecules, accelerating dehydration of the PEDOT:PSS film. In contrast, under high RH of 92%, adaptation appears slightly later because abundant water vapor slows water loss from the PEDOT:PSS layer. Nevertheless, within the preset operation window (7.5 s), substantial water desorption occurs under illumination for all humidity conditions, resulting in nearly identical final current. These results confirm that the TiO₂/PEDOT:PSS photomemristor can operate reliably across a wide range of environmental humidity levels. The weak dependence on ambient humidity is attributed to the spontaneous adsorption of water molecules by the hygroscopic PEDOT:PSS film, which reaches near-saturation under normal RH.

In situ infrared (IR) spectroscopy of TiO₂/PEDOT:PSS shows an absorption band at 3000–4000 cm⁻¹ (Fig. 4f), which corresponds to O–H stretching vibrations of water molecules^40,41^. The peak of this absorption band progressively decreases with temperature and almost disappears at 120 °C, indicating substantial dehydration of PEDOT:PSS. This finding further supports the aforementioned hypothesis regarding thermal-induced degradation of PEDOT:PSS. Furthermore, thermogravimetric analysis (TGA) of TiO₂/PEDOT:PSS also reveals pronounced weight loss (4.38%) before reaching 100 °C (Fig. 4g), which can be attributed to the water desorption. Complementary variable-temperature Fourier transform infrared (FT-IR) spectra of PEDOT:PSS show a remarkably decreased strength at 1175 cm^−^¹ that corresponds to the hydrophilic aromatic sulfonic group (i.e., C-O-C) after thermal annealing at 120 °C (Fig. S15). The decreased hydrophilicity correlates with the diminished O–H peak from water absorption^45,46^. Compared with the intact PEDOT:PSS film, the one after thermal annealing at 120 °C shows an increased contact angle from 60° to 66°, which also reveals reduced hydrophilicity (Fig. S15). Atomic force microscopy (AFM) measurements of PEDOT:PSS films at different temperatures show negligible change of surface roughness (Fig. S16), which excludes its influence on the light adaptation of the TiO₂/PEDOT:PSS-based photomemristor. Collectively, these results confirm the significant influence of temperature on the surface chemistry and conductivity of PEDOT:PSS via the regulation of water absorption. The light adaptation, especially the remarkably suppressed PSC, originates from the dominating photothermal effect over the photocurrent.

### Image recognition of TiO₂/PEDOT:PSS-based artificial visual systems in mixed-light conditions

**Fig. 5:**
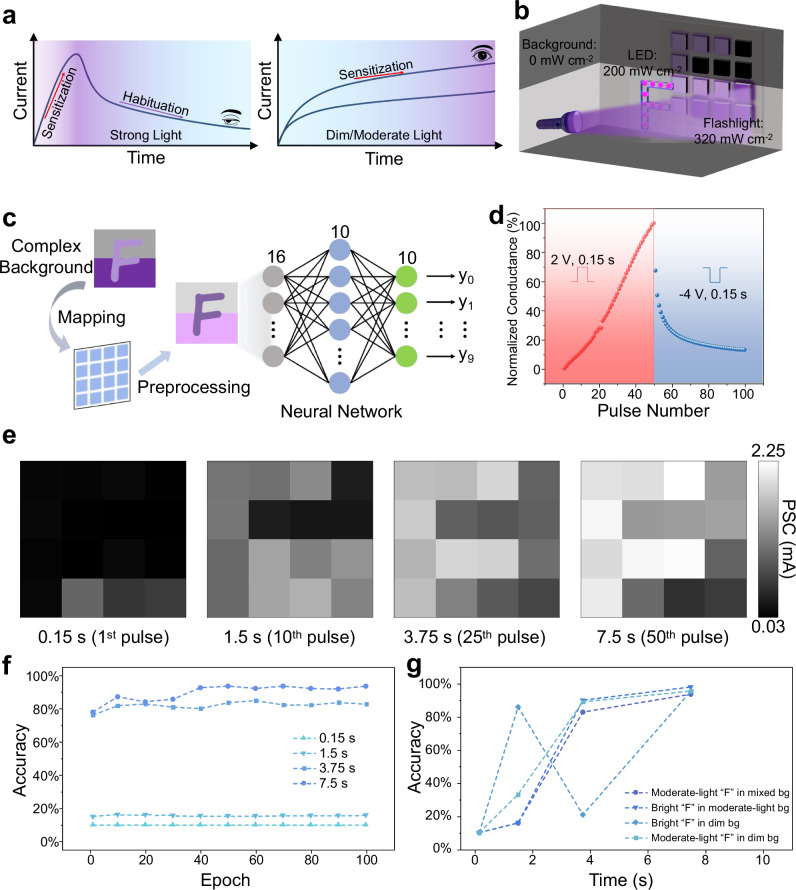
Artificial visual systems based on the photomemristor array combined with a neural network in different illumination conditions. **a** Schematic illustrating the photopic and scotopic adaptation of human visual systems under bright (left) and dim (right) conditions. **b** Experimental setup of the image recognition by the artificial visual system in mixed-light environments. **c** Working flow of the artificial visual system for image recognition. **d** Potentiation (red) and depression (blue) properties of the TiO₂/PEDOT:PSS-based photomemristor under 50 consecutive positive or negative voltage pulses. **e** Evolution of the image (i.e., “F”) captured by the artificial visual system at different time lapses (or pulse numbers). (*V*_bias_ = 1.5 V, *P*_w_ = 100 ms, *Δt* = 50 ms). **f** Prediction accuracy of the artificial neural network (ANN) fed by the image detected by the photomemristor array as a function of epochs. **g** Image recognition accuracy of the artificial visual system at different time lapses in different light environments.

Despite widely reported light-adaptation capabilities in the previous works^14–16,22,32,34,35^, they are only suitable for monochromatic environments. However, accurate image recognition in mixed-light conditions is essential for high-performance artificial visual systems during practical use in daily life. The large difference of PSC in dim and bright lights prevents conventional light-adaptive photomemristors from accurately detecting objects in mixed-light environments due to their limited PSC suppression in bright light, manifested by the loss of half the information. Although various algorithms and customized parameter tuning have been employed to recover lost information, postprocessing significantly constrains their practical efficiency and real-time image recognition. The TiO₂/PEDOT:PSS photomemristor developed in this work exhibits unprecedented adaptive behavior with a remarkably suppressed PSC that even falls below the dark-current level. Therefore, the remarkable light-adaptation of the TiO₂/PEDOT:PSS photomemristor can be leveraged to realize image recognition in mixed-light environments without using complex algorithms. In a proof-of-concept demonstration, a high-adaptive visual detection system based on a 4 × 4 photomemristor array is introduced to afford image recognition in mixed-light environments (Fig. 5a). An artificial neural network (ANN) is incorporated into the highly adaptive 4 × 4 photomemristor array to form the artificial visual system (Fig. 5c). To examine the uniformity of the photomemristor array, the PSC of sixteen units under a 1.5 V pulse voltage in three illumination conditions—dim (0 mW cm^−^², Fig. S17a), moderate (200 mW cm^−^², Fig. S17b), and bright (320 mW cm^−^², Fig. S17c) light is measured and compared. Despite variations of PSC, all units show remarkably suppressed postsynaptic current and light adaptation with the same inflection point in bright light (Fig. S17d). The following procedures are adopted in image recognition: PSC acquisition, neuromorphic preprocessing and training, and prediction. The synaptic parameters obtained under positive (2 V) and negative (-4 V) voltage pulses are fed to the ANN for training (Fig. 5d). The good linearity of spike-timing-dependent plasticity in the TiO₂/PEDOT:PSS photomemristor is beneficial for improving prediction accuracy. The performance of the artificial visual system is evaluated with five standard letter patterns, showcasing a high accuracy of over 90% after only 7 iterations (Fig. S18).

In the experimental setup, a moderately illuminated letter-patterned LED (200 mW cm^−^²) is positioned in front of a dim background (0 mW cm^−^²) in the upper panel and a bright light source (320 mW cm^−^²) in the lower panel (Fig. 5b) to emulate the mixed-light environment in daily life^47,48^. For example, a driver in the car needs to discern the traffic light (i.e., moderate brightness) and pedestrians under the high beam from the other car (i.e., bright light source) for a safe drive. The PSC of each photomemristor unit in the array upon voltage pulses (*V*_bias_ = 1.5 V, *P*_w_ = 0.15 s) is recorded at different time lapses (Fig. 5e). The photomemristor units that are exposed to the bright background show the highest PSC at 0.15 s (i.e., the 1st pulse). The PSC of units that are exposed to the bright and moderate light increases more significantly with time compared to those exposed to the dim light due to the contribution of photocurrent. With further increased time over 3.75 s, the PSC of the units exposed to the bright light decreases dramatically due to the remarkable suppression, while the PSC of those exposed to the moderate light (i.e., the “F” region) increases. As a result, the contrast of the PSC mapping by the artificial visual system continuously enhances with the increasing time lapse, leading to the discernible “F” pattern. The PSC mapping at different time lapses is fed into the ANN for the automatic image recognition. The improved recognition accuracy is achieved by exploiting the light adaptation of the photomemristor. For example, the recognition accuracy of the ANN fed by the PSC mapping at 0.15 s remains low (~ 10%) regardless of interactions. With the time increased to 7.5 s to trigger the light adaptation, the recognition accuracy increases significantly to 93.7% after 100 iterations (Fig. 5f). Besides the mixed-light environment, the artificial visual system also allows for image recognition in monochromatic environments, including bright objects in moderate-light conditions (Fig. S19a), and bright (Fig. S19b) and moderate-lighted (Fig. S19c) objects in dim conditions. Due to the remarkable PSC suppression, the “F” pattern shows a decreasing gray level, which becomes even lower than that corresponding to the dark background (Fig. S19b). Despite the overlap between the dark current and the suppressed PSC in bright light, the ANN is capable of recognizing the image with high accuracy (>95%) by applying more pulses (Fig. S20). Furthermore, as the training iteration increases, the fluctuation in recognition accuracy is gradually reduced to result in a smoother evolution of the accuracy curve, which demonstrates the high reliability of the proposed neural network. Overall, the artificial visual system combined with the ANN can afford accurate image recognition in both monochromatic and mixed-light conditions with a process time of 7.5 s (Fig. 5g).

## Discussion

In summary, this work presents a TiO₂/PEDOT:PSS-based photomemristor with vision adaptation to afford accurate image recognition in a mixed-light environment. Inspired by the bleaching and regeneration of rhodopsin in human eyes, the dynamic absorption and desorption of water on PEDOT:PSS change the resistance of the photomemristor via H₃O⁺ to modulate the optoelectrical conversion of TiO_2_. This leads to unprecedented suppression of the synaptic current in bright light, which is even lower than the dark current. Combined with an artificial neural network, the photomemristor array can achieve high accuracy in image recognition (~91.3%) under complex mixed-background conditions by exploiting the unprecedented adaptation. This work establishes a new approach for designing autonomous, energy-efficient, and high-performance neuromorphic optoelectronics to serve as the next-generation machines or robot vision systems in dynamic and unpredictable real-world illumination environments.

## Methods

### Materials

Tetrabutyl titanate was purchased from Aladdin (Shanghai Aladdin Biochemical Technology Co., Ltd., Shanghai, China). Diethanolamine was obtained from Energy Chemical (Anhui Zesheng Technology Co., Ltd., Anhui, China). PEDOT:PSS (Clevios PH1000) was provided by Xi’an Polymer Light Technology Co., Ltd. All chemicals were used without further purification.

### Preparation of TiO₂/PEDOT:PSS-based photomemristors

Fluorine-doped tin oxide (FTO) glass substrates (170 nm thick) were employed as the bottom electrodes. The FTO substrates were sequentially cleaned by ultrasonication in acetone, ethanol, and deionized (DI) water for 15 min each, followed by oxygen plasma treatment for 10 min to enhance surface hydrophilicity. The TiO₂ precursor solution was prepared by mixing diethanolamine (2.45 mL, 99.7%) and tetrabutyl titanate (8.5 mL, 99.0%) with ethanol (33.6 mL, 99.9%) under stirring for 2 h. Subsequently, a mixture of DI water (0.45 mL) and ethanol (5 mL) was added dropwise, followed by stirring for an additional 2 h to form a homogeneous TiO₂ sol–gel. The sol–gel was left still for 24 h at room temperature before use. The TiO₂ sol–gel was spin-coated onto the pre-cleaned FTO substrates at 3000 rpm for 30 s, followed by annealing at 450 °C for 1 h in ambient air. After cooling to room temperature, the TiO₂ films were treated with oxygen plasma for 1 min to further improve surface wettability before subsequent deposition.

A commercially available PEDOT:PSS aqueous dispersion was spin-coated onto the TiO₂/FTO substrates at 3000 rpm for 30 s. The resulting films were immediately transferred to a vacuum oven and thermally annealed at 120 °C for 1 h. Top circular electrodes (500 μm in diameter) were fabricated by magnetron sputtering of Ti/Au bilayers (5 nm/80 nm) and then patterned by standard photolithography.

### Characterization

All electrical characterizations were conducted under ambient conditions. Photoresponse and synaptic characteristics were measured using a Keysight B1500A semiconductor parameter analyzer. A pulse width (*P*_w_) of 100 ms and a time interval (*Δt*) of 50 ms were employed for all measurements, unless specified otherwise. Optical stimulation was provided by a commercial 365 nm LED light source, and the illumination intensity was calibrated with a standard optical power meter. The surface morphologies of the thin films were characterized using scanning electron microscopy (SEM, ZEISS Sigma 360) and atomic force microscopy (AFM, Bruker Dimension Icon) in tapping mode. The Raman spectroscopy was tested using the Renishaw inVia at 532 nm. The optical absorption of the films was characterized by an ultraviolet-visible spectrophotometer (PerkinElmer Lambda 35). The i*n situ* infrared spectroscopy was performed with the Bruker VERTEX 80 V.

## Supplementary information

Supplementary information

Description of Additional Supplementary Information

Supplementary Movie 1

Transparent Peer Review file

## Source data

Source Data

## Data Availability

Source data are provided with this paper. Additional data are available from the corresponding author upon request.

## Code availability

All code used in simulations supporting this article is available from the corresponding authors upon request.
