# Supplementary material for: Full vision adaptation in mixed-light conditions enabled by dynamic water adsorption/desorption
Source: Nat Commun. 2026 Jun 9;17:4965. doi: 10.1038/s41467-026-73217-7 (PMC13249880; doi:10.1038/s41467-026-73217-7)
Supplement: Supplementary file 1 — Supplementary information [file 41467_2026_73217_MOESM1_ESM.docx]

**Supplementary Information: Full Vision Adaptation in Mixed-light Conditions via Dynamic Water Adsorption/Desorption**

*Jia Zhu^1,2^* *^*^, Wantao Liu^1^, Wanxin Huang^3,4^, Xiangjie Chen^1^, Xuewei Feng^5^, Xin Luo^6,7^， Kai Xu^8^, Min Gao^1^, Haifeng Ling^4^, Chaoyun Song^8 *^ , Huanyu Cheng^9 *^, Yuan Lin^1,10 *^*

^1^ School of Materials and Energy, University of Electronic Science and Technology of China, Chengdu 610054, China

^2^ Yangtze Delta Region Institute (Quzhou), University of Electronics Science and Technology of China, Quzhou 324000, China

^3^ School of Automation and Engineering, University of Electronic Science and Technology of China, Chengdu 610054, China

^4^ Nanjing University of Posts & Telecommunications (NJUPT), Nanjing, 210023 China

^5^ School of Mechanical Engineering, Shanghai Jiao Tong University, Shanghai 200240, China

^6^ Guangdong Provincial Key Laboratory of Magnetoelectric Physics and Devices, School of Physics, Sun Yat-sen University, Guangzhou 510275, China

^7^ State Key Laboratory of Optoelectronic Materials and Technologies, School of Physics, Sun Yat-sen University, 510275, Guangzhou, China.

^8^ Department of Engineering, King’s Conewllege London, London WC2R 2LS, UK

^9^ Department of Engineering Science and Mechanics, The Pennsylvania State University, University Park, Pennsylvania 16802, USA.

^10^ Medico-Engineering Cooperation on Applied Medicine Research Center, University of Electronics Science and Technology of China, Chengdu 610054, China

^*^To whom correspondence should be addressed. E-mail: zhujia1990@uestc.edu.cn (J.Z.), Huanyu.Cheng@psu.edu (H.C.), linyuan@uestc.edu.cn (Y.L.), and chaoyun.song@kcl.ac.uk (C.S.).

**
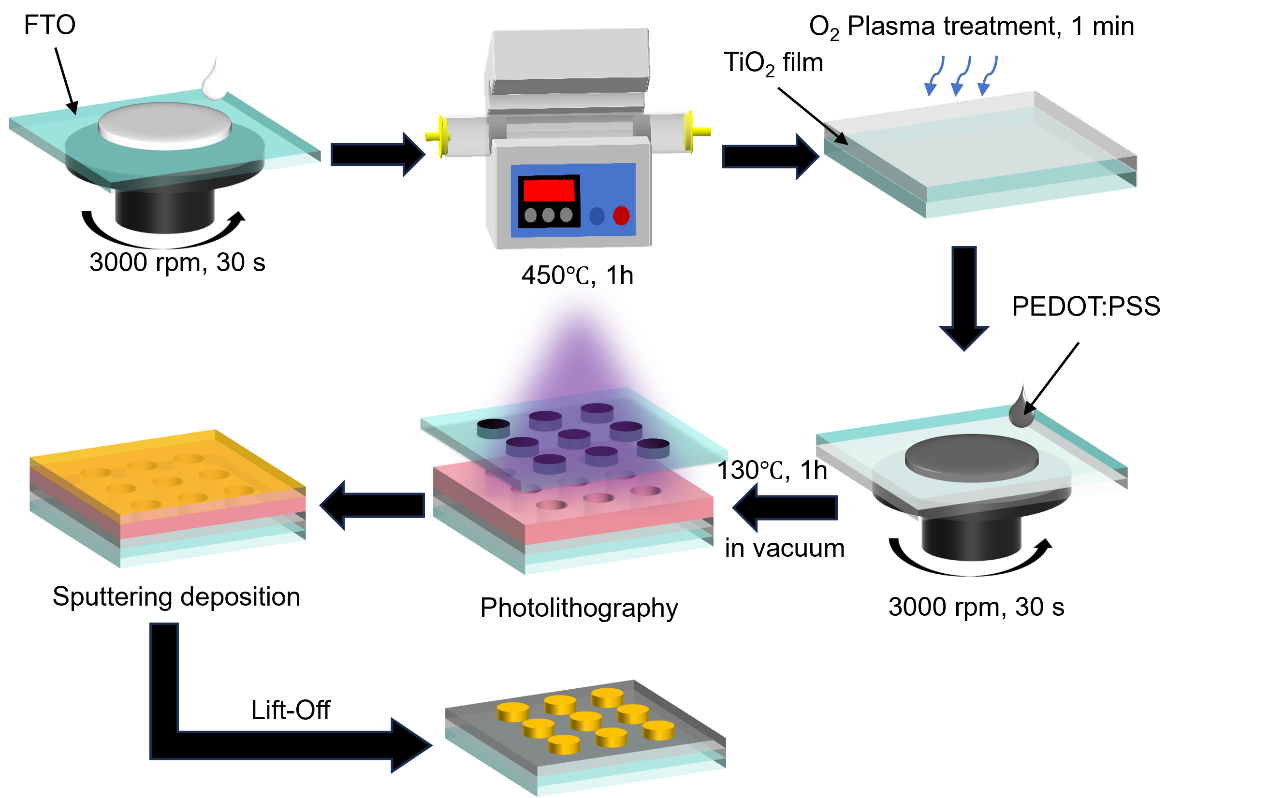
**

**Fig. S1. Fabrication process of the adaptative photomemristor.** Schematics illustrating the fabrication process of the TiO₂/PEDOT:PSS-based photomemristor.


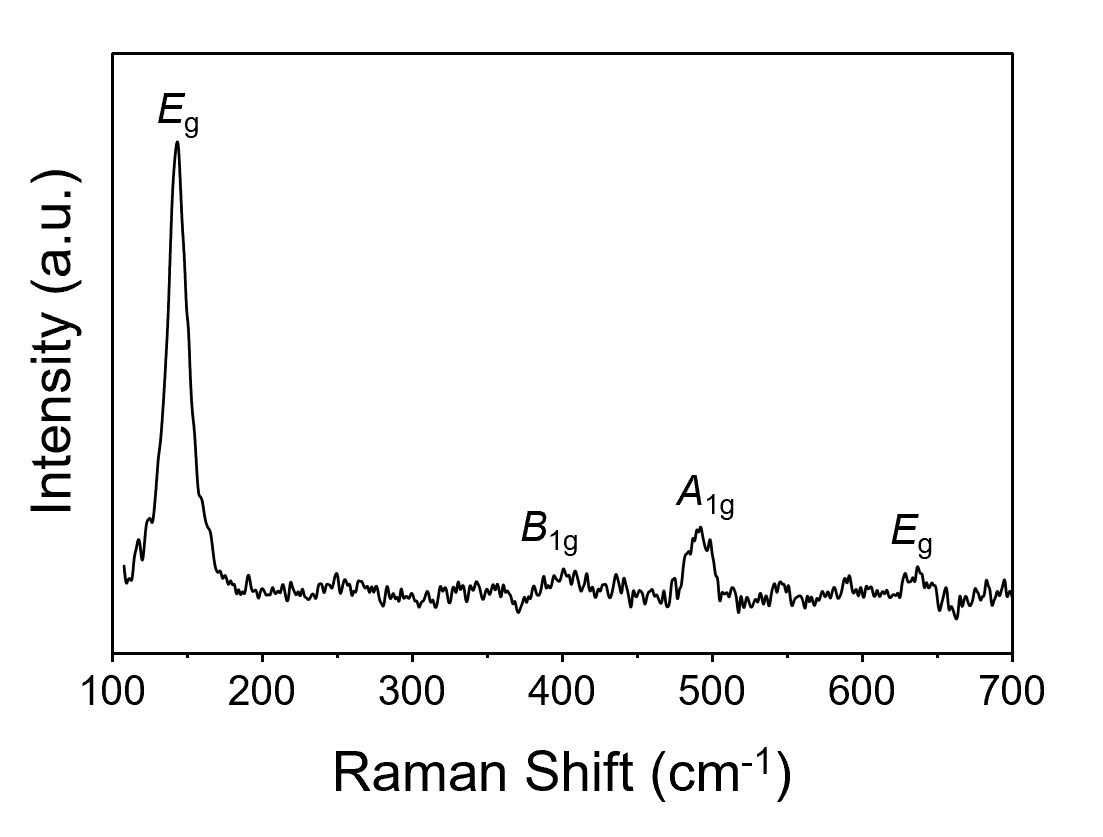


**Fig. S2. Characterization of crystal structures of TiO₂ thin film.** Raman spectrum of the TiO₂ film. The characteristic peaks at approximately 143 cm⁻¹, 399 cm⁻¹, 492 cm⁻¹, and 633 cm⁻¹ are attributed to the *E*_g_, *B*_1g_, *A*_1g_, and *E*_g_ vibrational modes of anatase TiO₂, respectively.


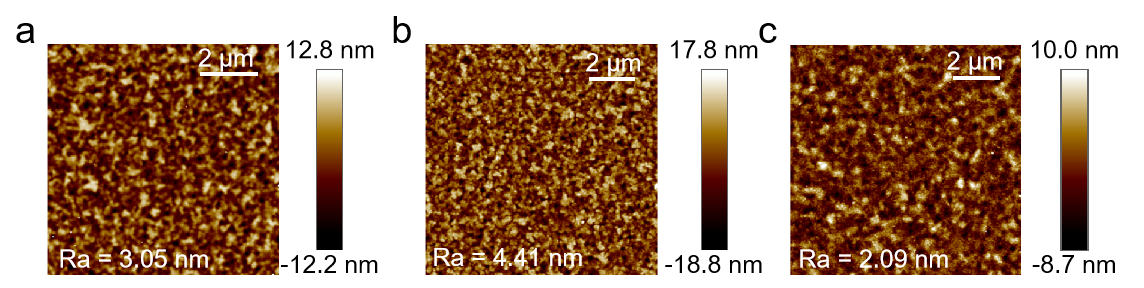


**Fig. S3.** **Surface Morphology Characterization by AFM.**  AFM images of the **(a)** TiO_2_, **(b)** PEDOT:PSS, and **(c)** TiO_2_/PEDOT:PSS film on FTO substrates.

**
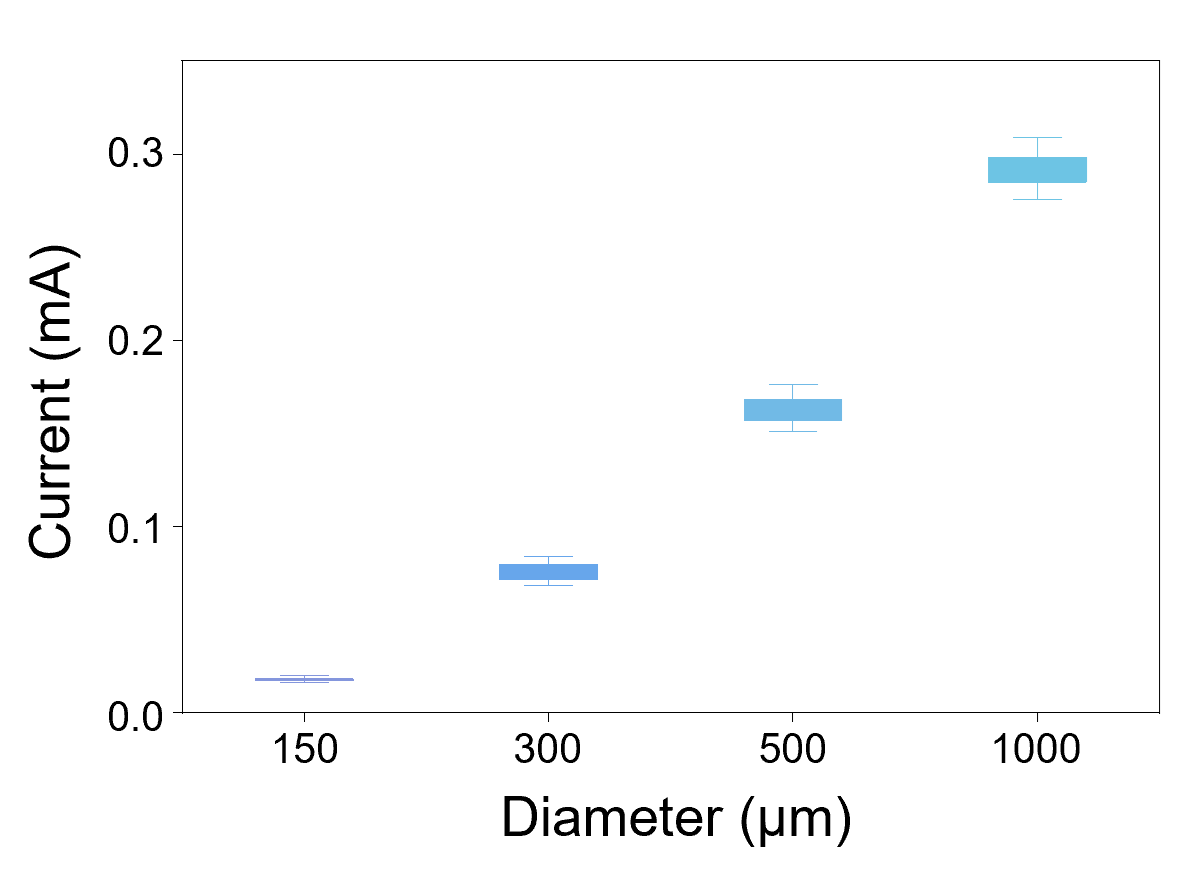
**

**Fig. S4. Size-dependence of the current in the TiO_2_/PEDOT:PSS-based photomemristor.** Current of the TiO₂/PEDOT:PSS-based photomemristor as a function of the units’ size at a bias voltage of 2 V. Data are presented as mean ± SD (n = 5 independent measurements).


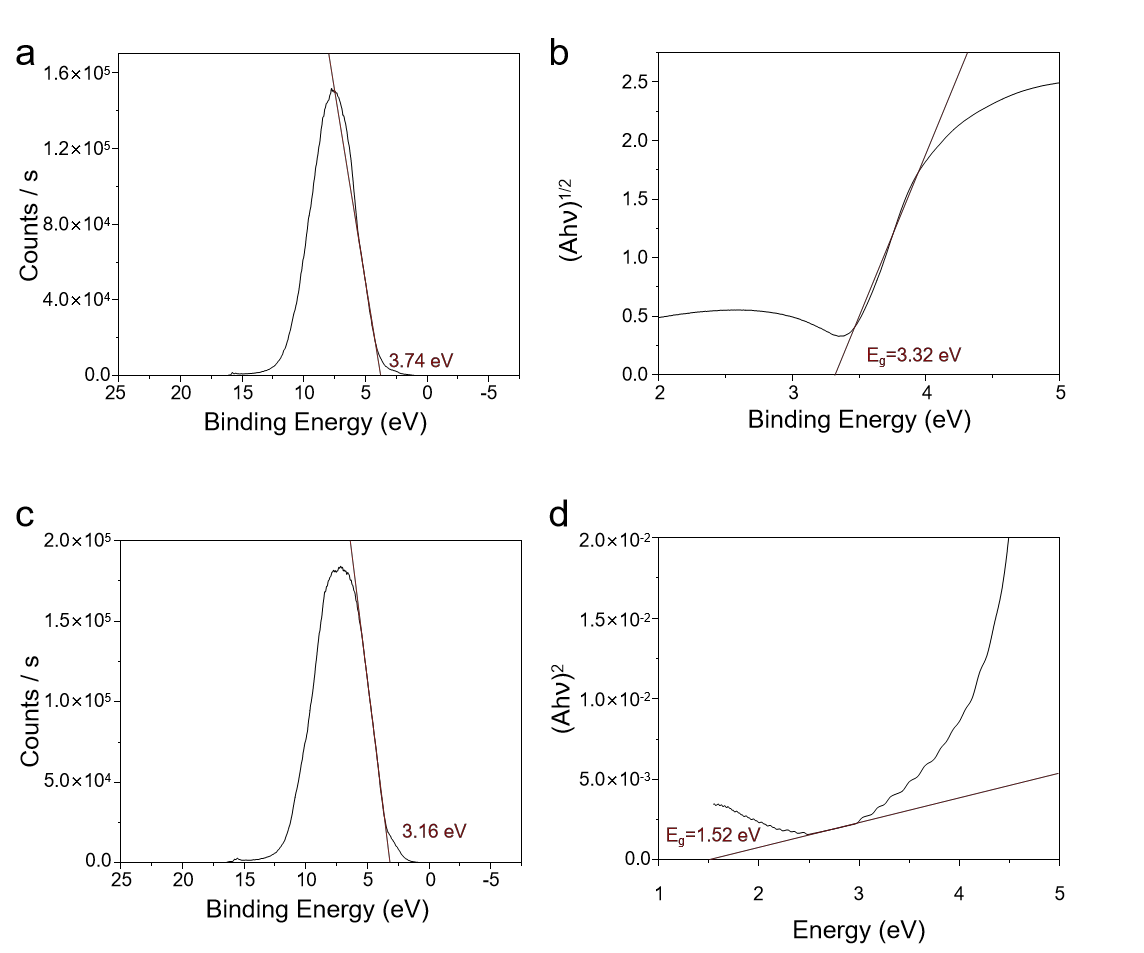


**Fig. S5. Band structure characterization of TiO₂ and PEDOT:PSS.** (**a**) Conduction band (C_B_) level of TiO₂ determined by ultraviolet photoelectron spectroscopy (UPS). (**b**) Tauc plot derived from the UV–vis absorption spectrum of TiO₂ for estimating the indirect bandgap. (**c**) The lowest unoccupied molecular orbital (LUMO) level of PEDOT:PSS determined by UPS. (**d**) Tauc plot derived from the UV–vis absorption spectrum of PEDOT:PSS for estimating the direct bandgap.


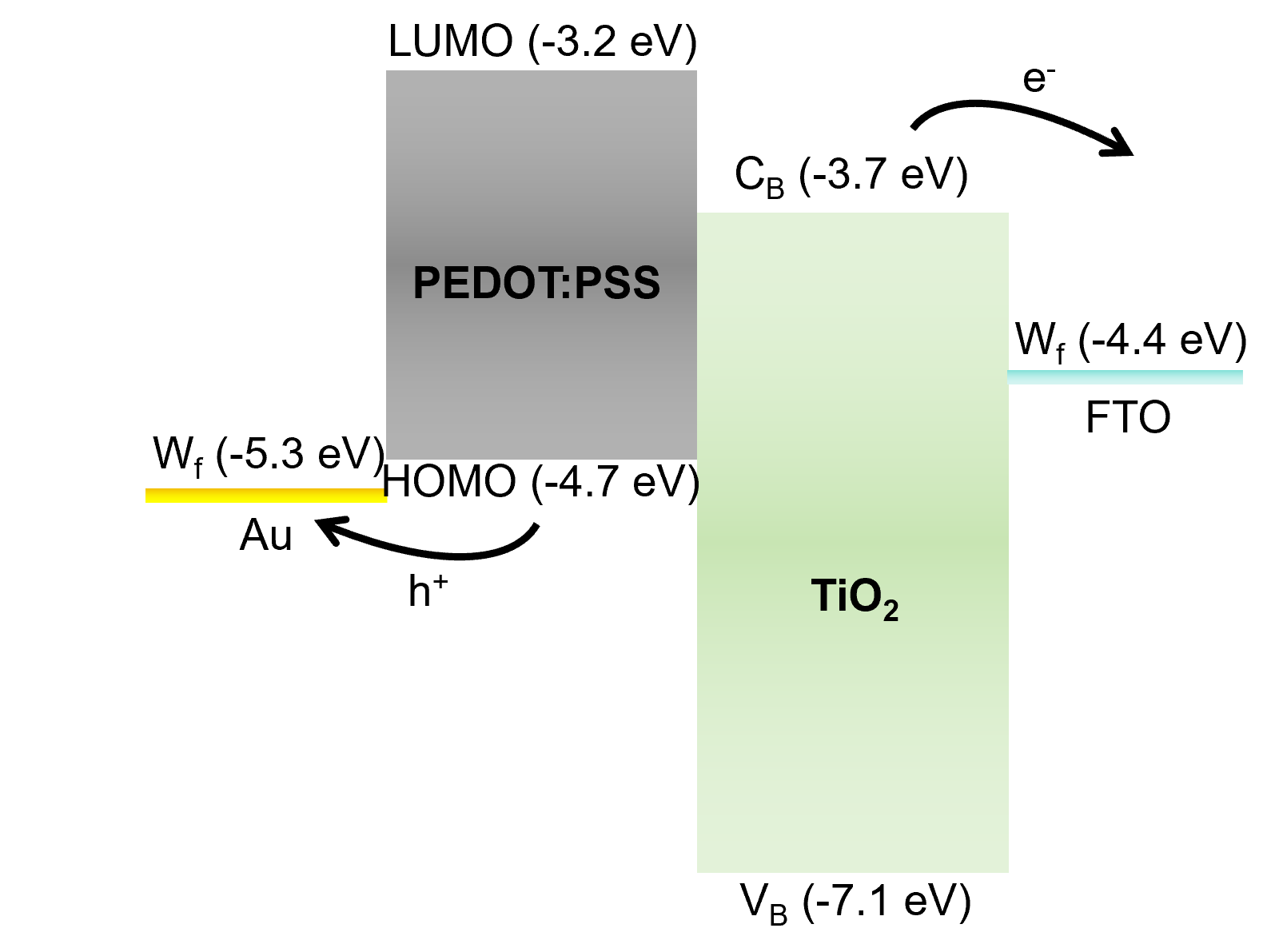


**Fig. S6.** **Energy band alignment of the photomemristor.** Schematic illustrating the energy band alignment of the TiO_2_/PEDOT:PSS photomemristor derived from the measured band structure of TiO_2_ and PEDOT:PSS.

**
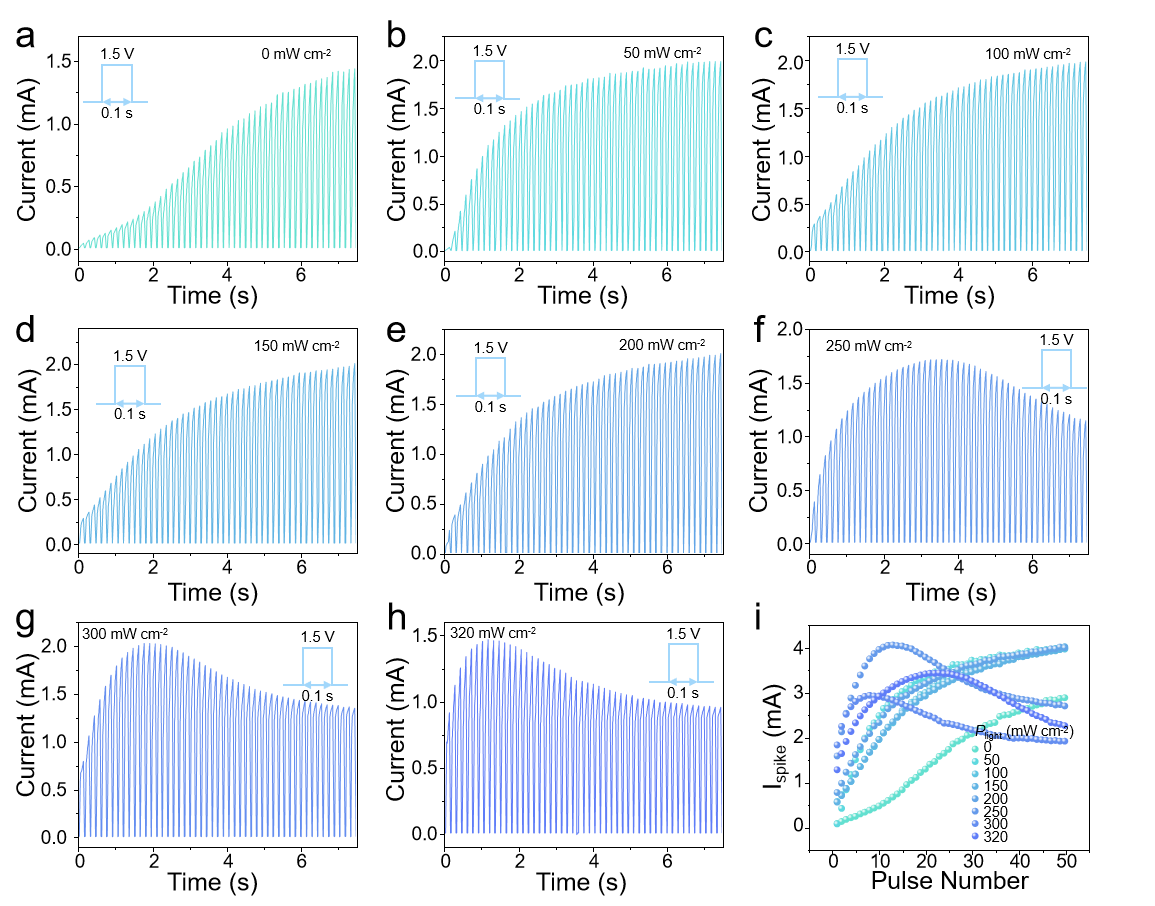
**

**Fig. S7.** **Plasticity of the TiO_2_/PEDOT:PSS-based photomemristor** **in different light intensities.** **(a**-**h)** Excitatory postsynaptic current (EPSC) responses of the TiO_2_/PEDOT:PSS-based photomemristor to 50 consecutive electrical pulses (*V*_bias_ = 1.5 V) upon different light intensities ranging from 0 to 320 mW cm^-2^. **(i)** Spike current response (*I*_spike_) of the TiO_2_/PEDOT:PSS-based photomemristor as a function of the pulse number in different light intensities. The pulse width (*P*_w_) is set to 100 ms, and the pulse interval (*Δt*) is set to 50 ms.

**
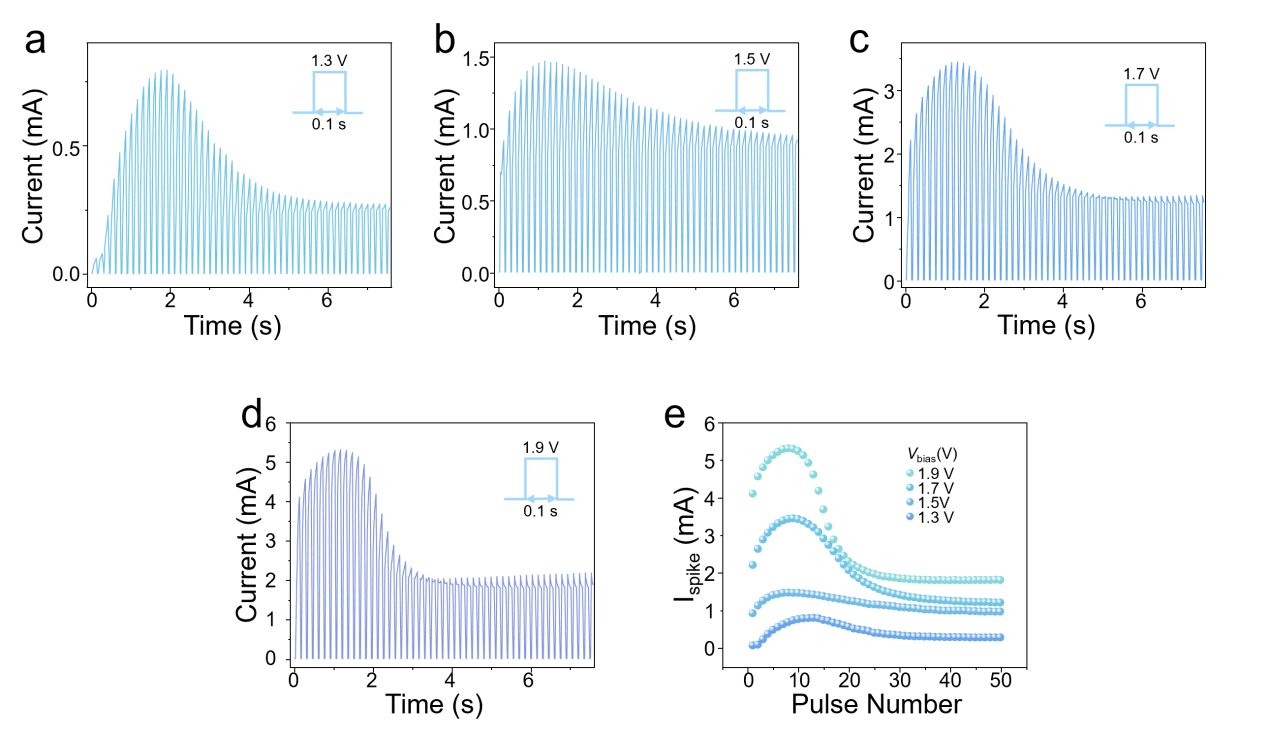
**

**Fig. S8.** **Plasticity of the TiO_2_/PEDOT:PSS-based upon the positive voltage pulse with different magnitudes.** **(a**-**d)** EPSC responses of the TiO_2_/PEDOT:PSS-based photomemristor to 50 consecutive electrical pulses with different amplitudes (*V*_bias_ = 1.3, 1.5, 1.7, 1.9 V) upon a light intensity of 320 mW cm^-2^. **(e)** *I*_spike_ of the TiO_2_/PEDOT:PSS-based photomemristor as a function of the pulse number with pulse amplitudes. *P*_w_ = 100 ms, Δ*t* = 50 ms.

**
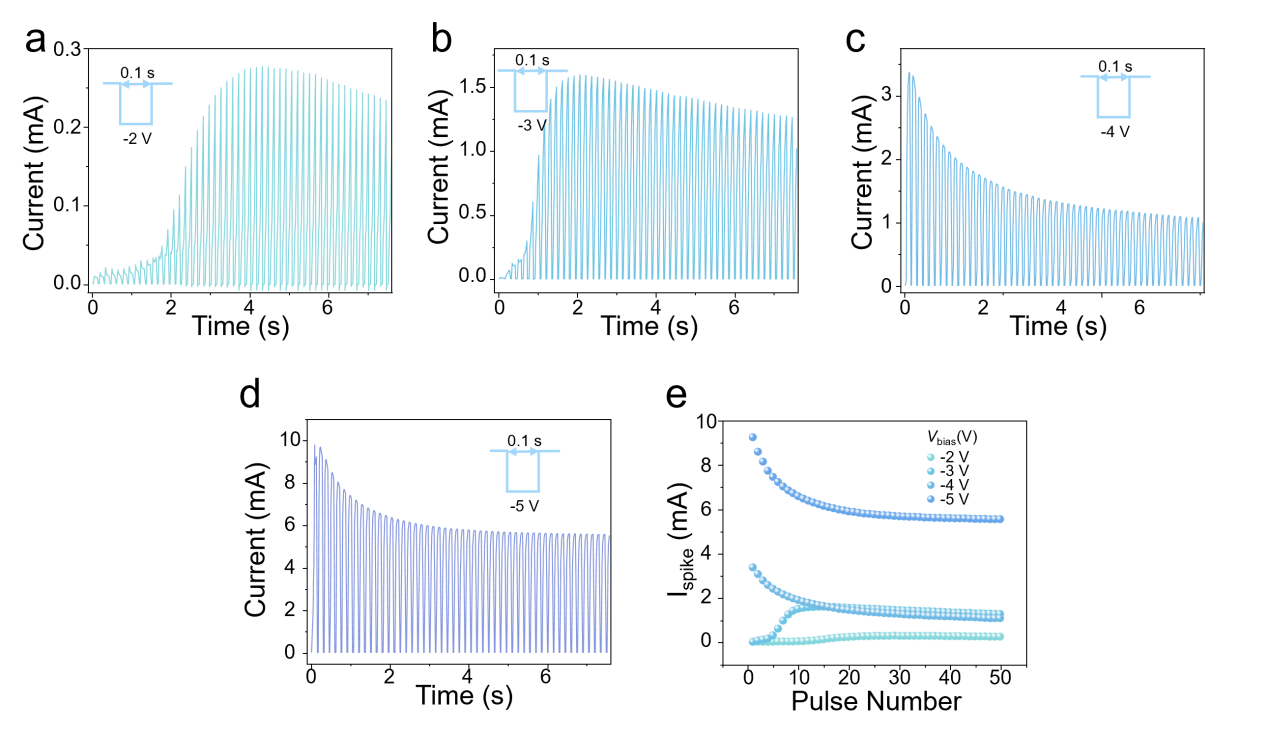
Fig. S9. Plasticity of the TiO_2_/PEDOT:PSS-based upon the negative voltage pulse with different magnitudes.** **(a**-**d)** IPSC responses of the TiO_2_/PEDOT:PSS-based photomemristor to 50 consecutive electrical pulses with different amplitudes (*V*_bias_ = -2, -3, -4, -5 V) upon a light intensity (*P*_light_) of 320 mW cm^-2^. **(e)** *I*_spike_ of the TiO_2_/PEDOT:PSS-based photomemristor as a function of the pulse number with pulse amplitudes. *P*_w_ = 100 ms, Δ*t* = 50 ms.


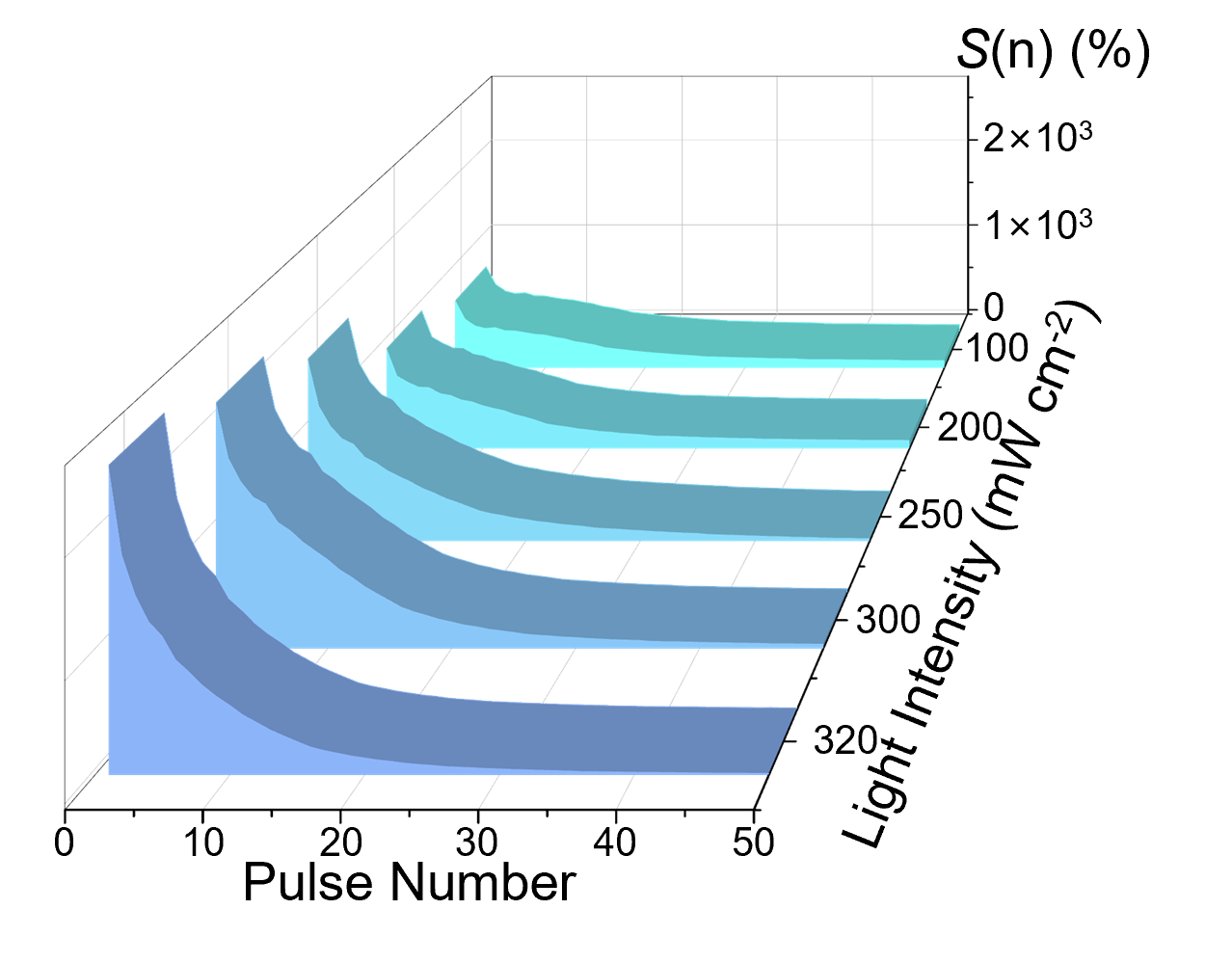


**Fig. S10.** **Adaptive performance of the TiO₂/PEDOT:PSS-based photomemristor under varying light pulse conditions and intensities.** Photosensitivity score (*S*(n)) as a function of pulse number at different light intensities. *V*_bias_ = 1.5 V, *P*_w_ = 100 ms, Δ*t* = 50 ms.


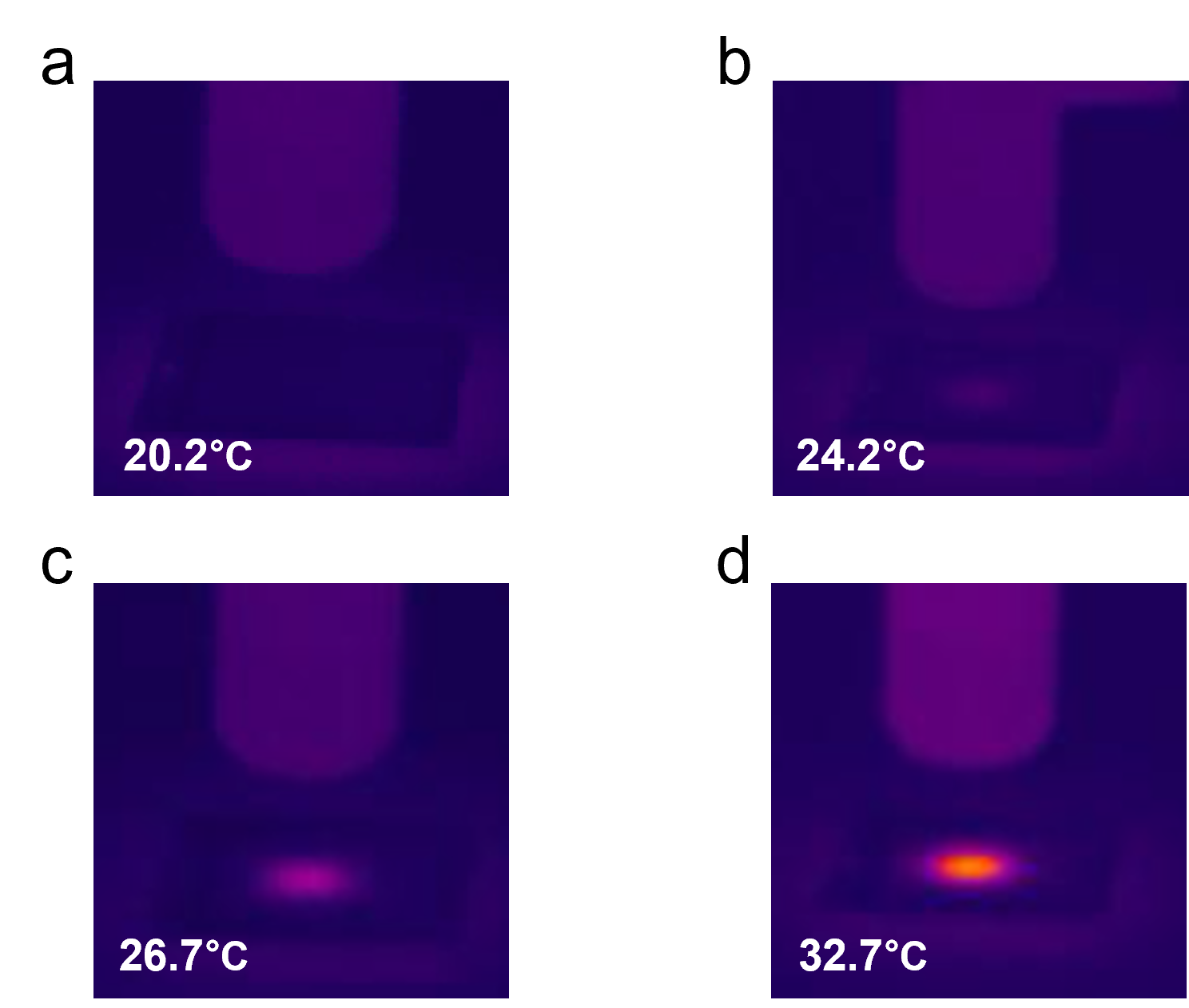


**Fig. S11. Photothermal effect in the TiO_2_/PEDOT:PSS-based photomemristor.** Infrared (IR) thermal images of the TiO_2_/PEDOT:PSS-based photomemristor **(a)** in the dark and **(b-d)** under 7.5 s of UV illumination with intensities of **(b)** 100, **(c)** 200, and **(c)** 320 mW cm^-2^ (365 nm), respectively.

**
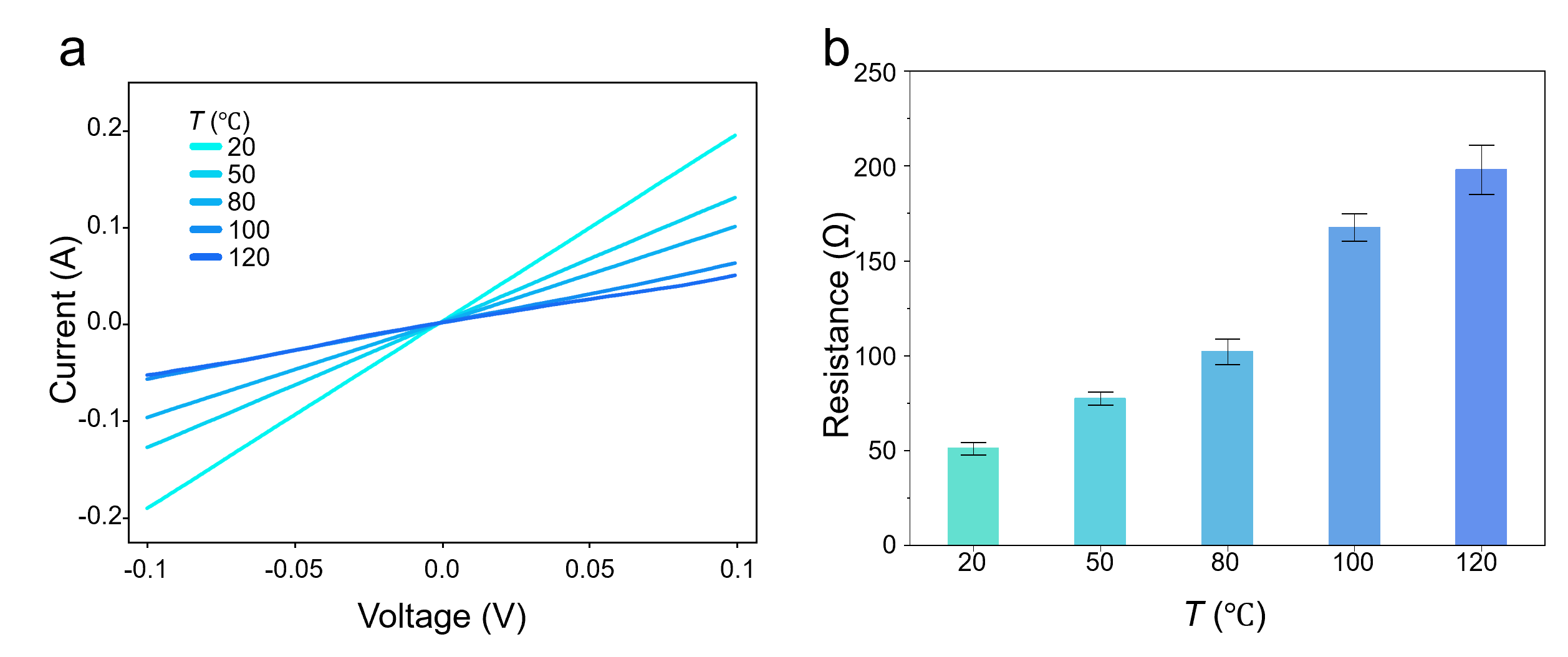
**

**Fig. S12.** **Temperature-dependent electrical conductivity of PEDOT:PSS films.** **(a)** *I-V* curves and **(b)** electrical resistance of PEDOT:PSS films extracted from the linear region of the *I-V* curves at different temperatures. Data are presented as mean ± SD (n = 8 independent samples).

**
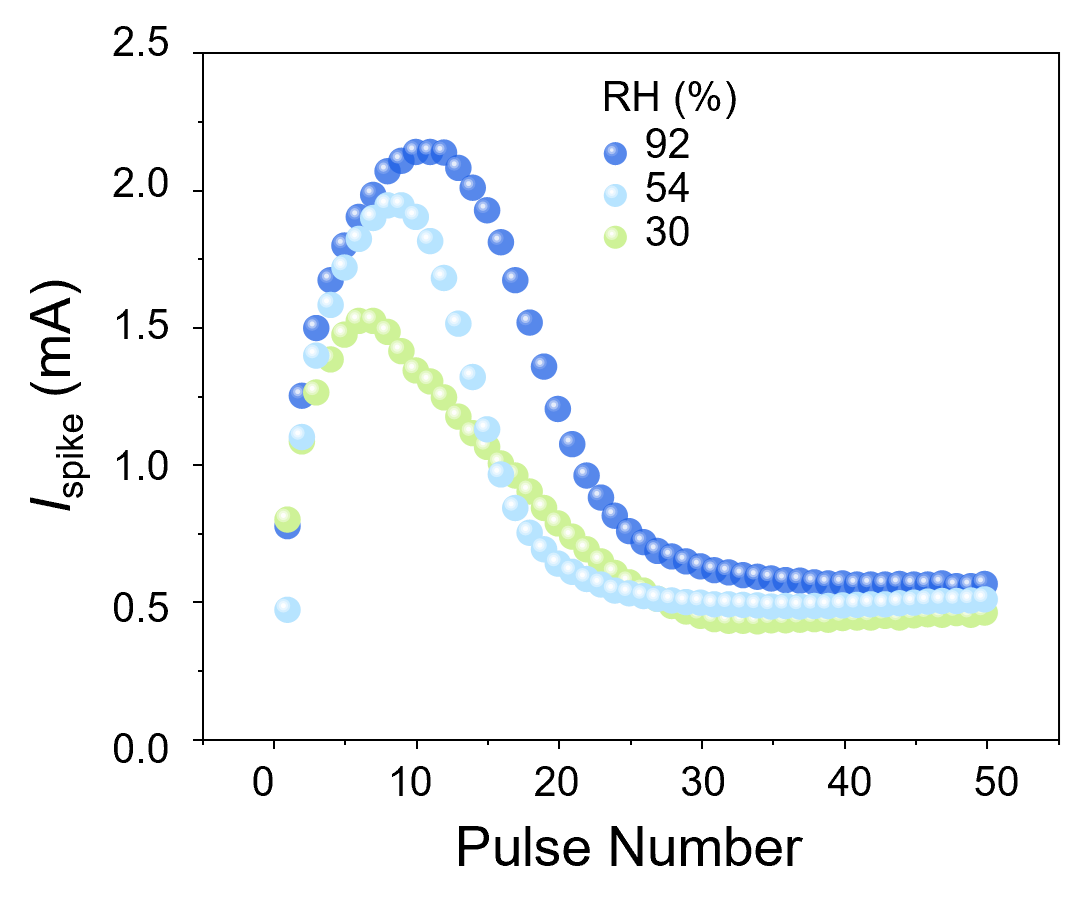
**

**Fig. S13. Influence of humidity on the adaptation performance of the TiO_2_/PEDOT:PSS-based photomemristor.** *I*_spike_ of the TiO_2_/PEDOT:PSS-based photomemristor under 320 mW cm^-2^ illumination at relative humidity (RH) levels of 92%, 54%, and 30%.


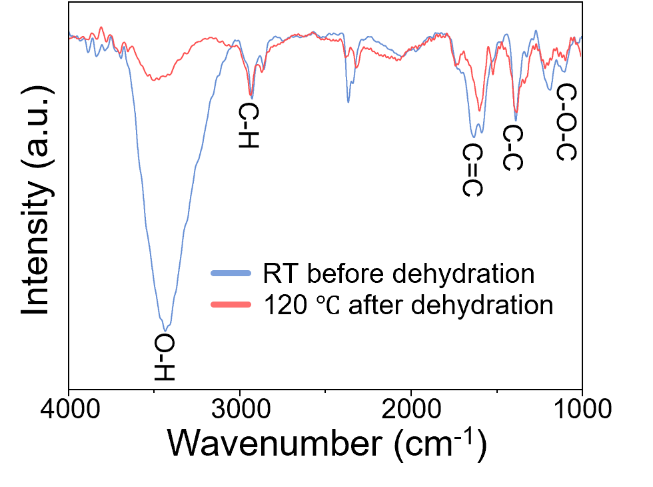


**Fig. S14.** **Influence of thermal annealing on the water content of the PEDOT:PSS film.**  Complementary variable-temperature Fourier transform infrared (FT-IR) spectra of the PEDOT:PSS film.


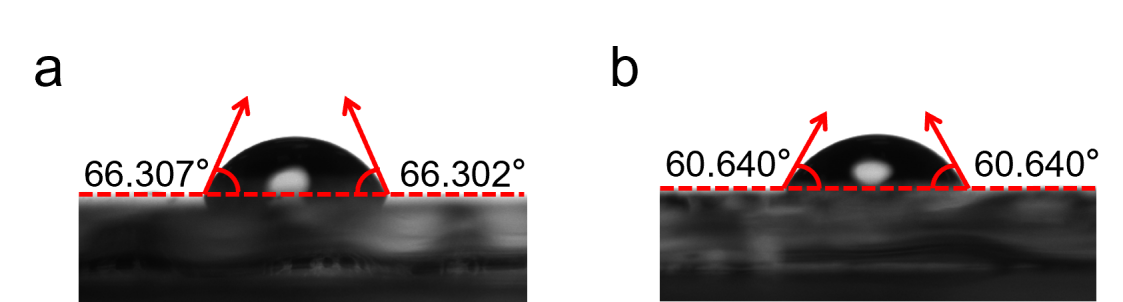


**Fig. S15.** **Influence of thermal annealing on the hydrophilicity of the TiO_2_/PEDOT:PSS bilayer film.** Water contact angles of the TiO_2_/PEDOT:PSS bilayer film **(a)** after dehydration by thermal annealing and **(b)** then rehydration by soaking in water.


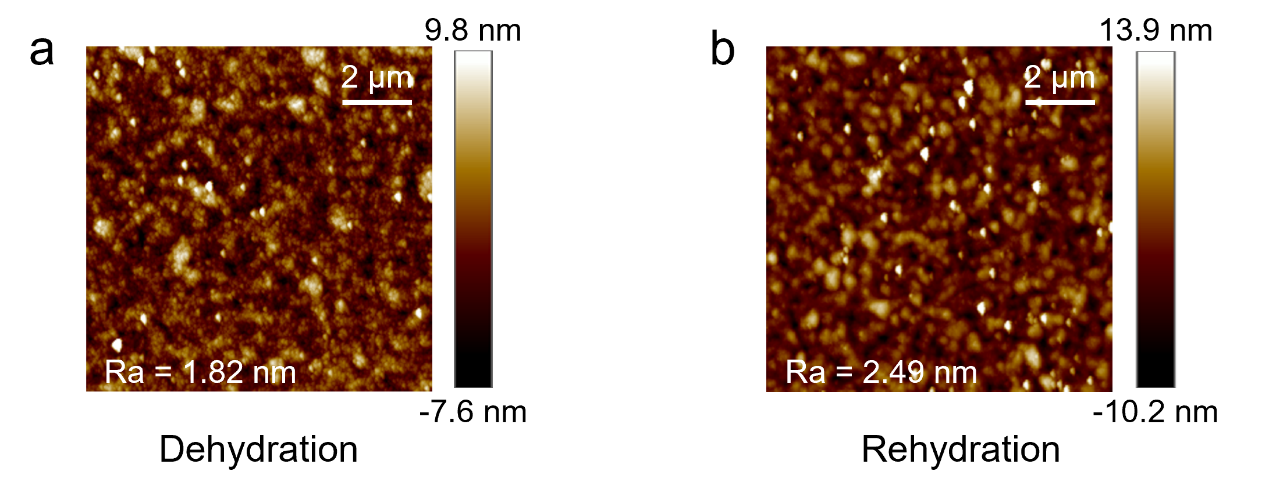
**Fig. S16. Influence of thermal annealing on the surface morphology of the TiO_2_/PEDOT:PSS bilayer film.**  AFM images of the TiO_2_/PEDOT:PSS bilayer film **(a)** after dehydration by thermal annealing and **(b)** then rehydration by soaking in water.


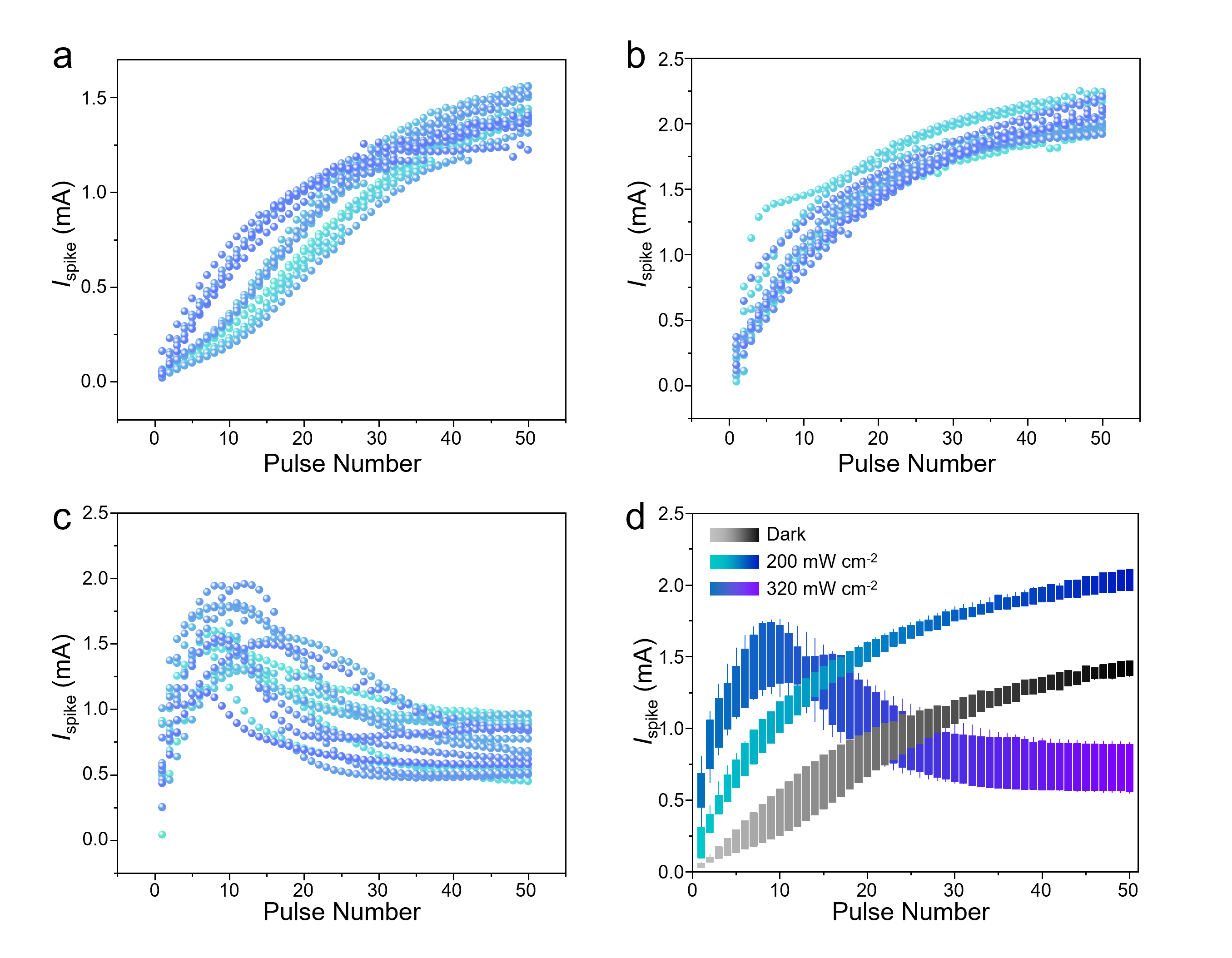


**Fig. S17.** **Sample-to-sample variations of TiO_2_/PEDOT:PSS-based photomemristors.** *I*_spike_ of 16 TiO_2_/PEDOT:PSS-based photomemristors as a function of the pulse number in different light intensities: **(a)** 0 mW cm^-2^, **(b)** 200 mW cm^-2^, and **(c)** 320 mW cm^-2^. **(d)** Statistics of *I*_spike_ in different light intensities. Data are presented as mean ± SD (n = 16 independent samples). *V*_bias_ = 1.5 V, *P*_w_ = 100 ms, Δ*t* = 50 ms.

**
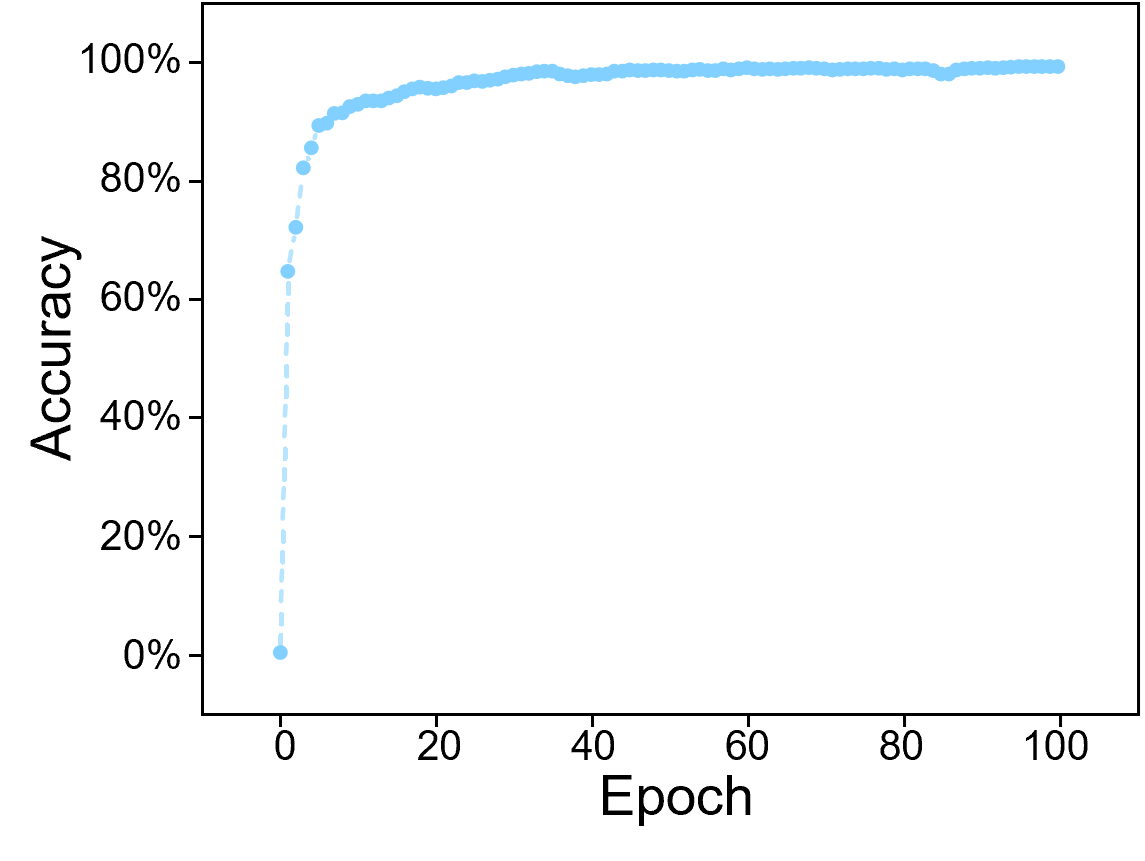
**

**Fig. S18.** **Artificial neural network (ANN) prediction accuracy versus training epochs.**  Prediction accuracy of the ANN fed by the image detected by five standard letter patterns (A, C, F, I, U, K, D, O, Z, L) as a function of epochs.

**
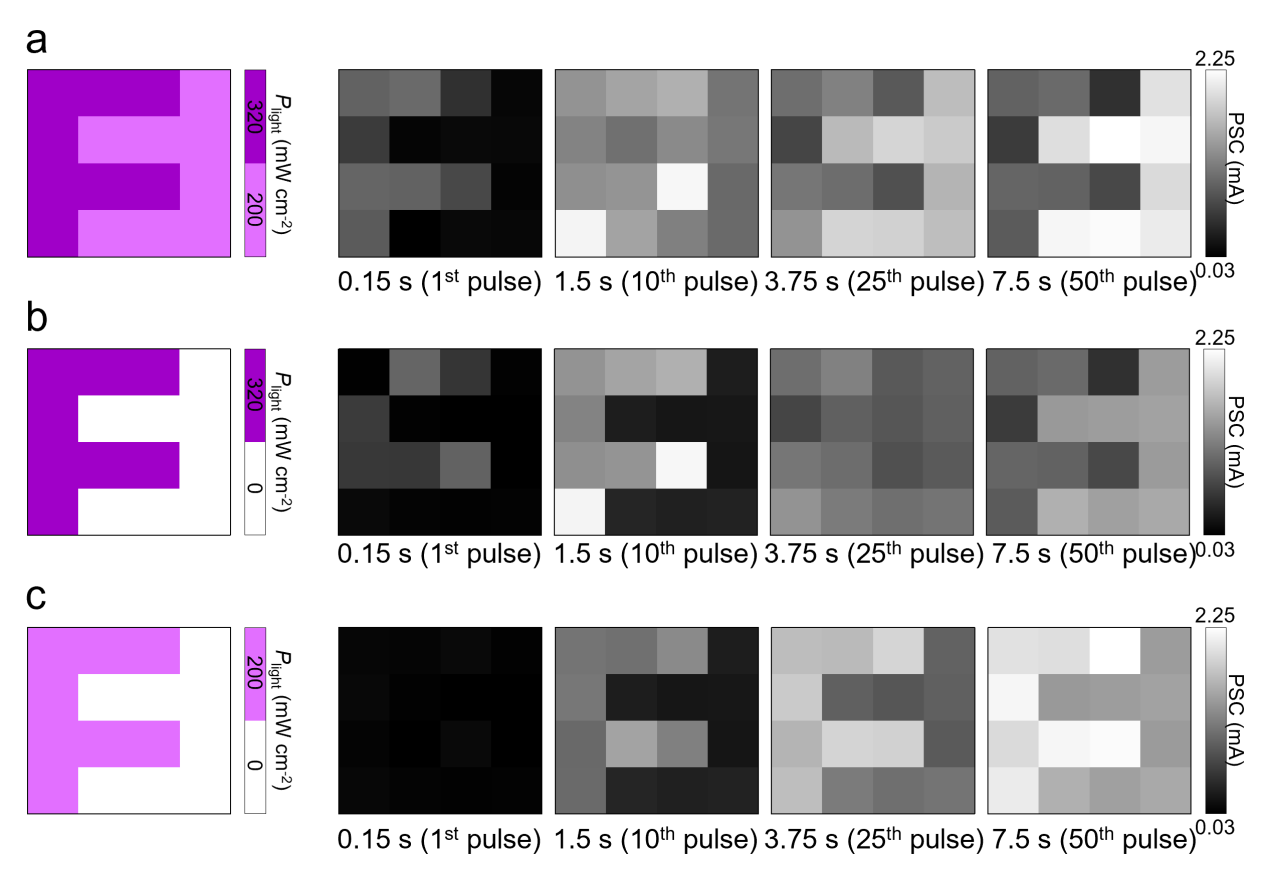
**

**Fig. S19. Dynamic imaging of letter patterns under varying illumination Conditions.** Artificial visual systems based on the photomemristor array combined with a neural network in different illumination conditions, including **(a)** bright “F” (320 mW cm⁻²) against a moderate-light background (200 mW cm⁻²), **(b)** bright “F” (320 mW cm⁻²) against a dim background (0 mW cm⁻²), and **(c)** Moderate-light “F” (200 mW cm⁻²) against a dim background (0 mW cm⁻²). The left panel shows the experimental setup. The right panel shows the image captured by the artificial visual system at different time lapses (or pulse numbers). *V*_bias_ = 1.5 V, *P*_w_ = 100 ms, Δ*t* = 50 ms.


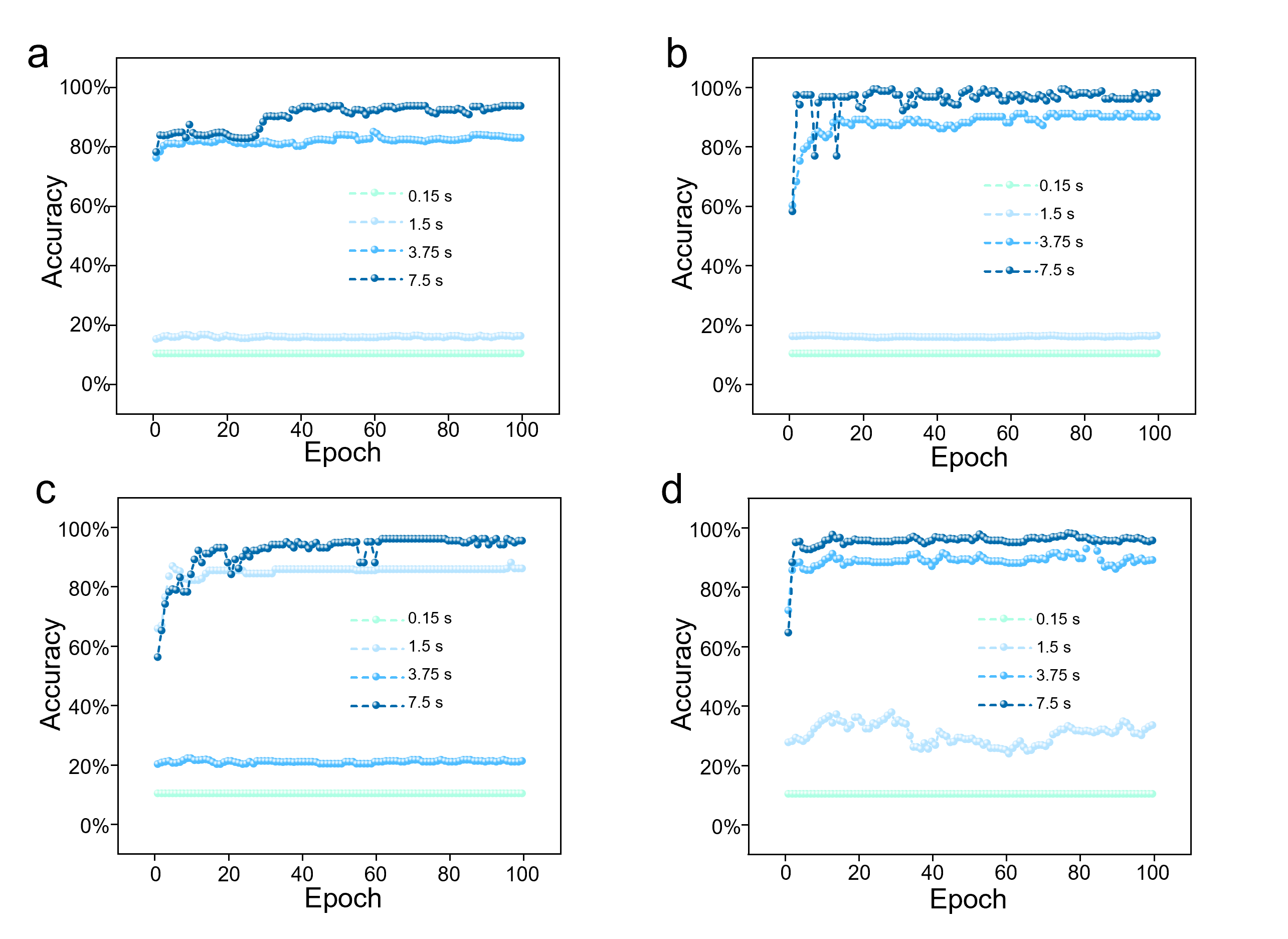


**Fig. S20. ANN prediction accuracy under varying illumination conditions.** Prediction accuracy of the ANN as a function of epochs in different illumination conditions, including (a) moderate-light “F” (200 mW cm−2) against a mixed-light background (0 and 320 mW cm−2 for the top and bottom panel), (b) bright “F” (320 mW cm−2) against a moderate-light background (200 mW cm−2), (c) bright “F” (320 mW cm−2) against a dim background (0 mW cm−2), and (d) moderate-light “F” (200 mW cm−2) against a dim background (0 mW cm−2). The postsynaptic current distribution (or images) detected by the array at different time lapses (i.e., 0.15, 1.5, 3.75, 7.5s) are fed to the ANN for training.

**Table S1.** Comparison between the TiO_2_/PEDOT:PSS-based photomemristor and the human visual system.

| **Parameter** | **This Work** | **Human vision system** |
| --- | --- | --- |
| Light adaptation | 1.5 s | ~5 min^1^ |
| Dark adaptation | <1 s | ~20-30 min^2^ |
| Power consumption | ~3.3 mW (max) | ~3 mW (retina)^3^ |
| Adaptation mechanism | Adsorption/desorption of H_2_O molecules | Rhodopsin bleaching–regeneration cycle |
| Structure | 2 functional layers | ~10 reinal layers^4^ |
| Thickness | ~120 nm | ~250 μm (retina)^5^ |

**Supplementary References:**

1. Peachey NS, Alexander KR, Derlacki DJ, Fishma GA. Light adaptation, rods, and the human cone flicker ERG. *Visual Neuroscience* **8**, 145-150 (1992).

2. Lamb TD, Pugh EN. Phototransduction, dark adaptation, and rhodopsin regeneration the proctor lecture. *Investigative ophthalmology & visual science* **47**, 5138-5152 (2006).

3. Harding CF. Brief alteration in dopaminergic function during development causes deficits in adult reproductive behavior. *Journal of neurobiology* **61**, 301-308 (2004).

4. Strachan JP, Strukov DB, Borghetti J, Joshua Yang J, Medeiros-Ribeiro G, Stanley Williams R. The switching location of a bipolar memristor: chemical, thermal and structural mapping. *Nanotechnology* **22**, 254015 (2011).

5. Read SA, Collins MJ, Vincent SJ, Alonso-Caneiro D. Macular retinal layer thickness in childhood. *Retina* **35**, 1223-1233 (2015).
