# Supplementary material for: Full vision adaptation in mixed-light conditions enabled by dynamic water adsorption/desorption
Source: Nat Commun. 2026 Jun 9;17:4965. doi: 10.1038/s41467-026-73217-7 (PMC13249880; doi:10.1038/s41467-026-73217-7)
Supplement: Supplementary file 2 — Description of Additional Supplementary Information [file 41467_2026_73217_MOESM2_ESM.pdf]

**Description of Additional Supplementary Files**

File Name: Supplementary Movie 1
Description: Infrared (IR) thermography measurements at four illumination intensities (0, 100, 200, and 320 mW $cm^{-2}$) at a constant room temperature of 20 °C quantitatively evaluate the photothermal effect under illumination for 7.5 s.
