# Supplementary material for: Full vision adaptation in mixed-light conditions enabled by dynamic water adsorption/desorption
Source: Nat Commun. 2026 Jun 9;17:4965. doi: 10.1038/s41467-026-73217-7 (PMC13249880; doi:10.1038/s41467-026-73217-7)
Supplement: Supplementary file 4 — Transparent Peer Review file [file 41467_2026_73217_MOESM4_ESM.pdf]

nature portfolio

Peer Review File

Full Vision Adaptation in Mixed-light Conditions Enabled by Dynamic Water Adsorption/Desorption

Corresponding Author: Professor Huanyu Cheng

This file contains all reviewer reports in order by version, followed by all author rebuttals in order by version.

Version 0:

Reviewer comments:

Reviewer #1

(Remarks to the Author)
This manuscript reports a TiO_2/PEDOT:PSS-based photomemristor capable of adaptive visual processing under mixed-light conditions, enabled by a light-modulated water adsorption/desorption process in the PEDOT:PSS layer. The device demonstrates pronounced synaptic current suppression under strong illumination—reaching levels even below the dark current—and, when implemented in a 4×4 array coupled with an artificial neural network, achieves a recognition accuracy of 91.3% under mixed-light scenarios. The study presents a promising bioinspired strategy for adaptive neuromorphic vision. While the results are compelling, several aspects of the experimental validation and mechanistic interpretation require clarification or additional support. Some revisions are therefore recommended to improve the rigor and completeness of the work. The following points should be addressed:
1. Quantitative benchmarking of bioinspired vision adaptation.
The analogy to human visual adaptation is currently discussed in a largely qualitative manner. To strengthen this claim, a quantitative comparison is recommended. Key adaptive metrics (e.g., dynamic range, adaptation time constants, suppression ratios) should be benchmarked against reported values for human photopic and scotopic vision, preferably summarized in a table or comparative figure.
2. Experimental validation of the oxygen-vacancy-based switching mechanism.
The attribution of memristive switching to oxygen vacancy migration, as discussed in Figure 2g, lacks direct experimental evidence. To substantiate this mechanism, it is recommended to examine the dependence of device current on electrode area. If bulk oxygen vacancy migration dominates conduction, the current should scale proportionally with electrode area. Area-dependent electrical measurements would provide critical support for this interpretation.
3. Quantification of photothermal effects under illumination.
The light-adaptive behavior is attributed to photothermal heating-induced water desorption in the PEDOT:PSS layer. However, the manuscript does not establish a direct quantitative relationship between illumination intensity and the resulting temperature rise at the device surface. Experimental measurements—such as infrared thermography—to map surface temperature distributions under different light intensities are strongly recommended.
4. Revision of resistance extraction methodology in Figure S8b.
The resistance analysis presented in Figure S8b is methodologically inappropriate. Averaging V/I values across different points on nonlinear I–V curves does not yield a physically meaningful resistance. Instead, resistance should be consistently extracted from each I–V trace, for example by calculating the inverse slope of the linear (ohmic) region. The resulting resistance values should then be statistically compared across temperatures and device samples.
5. Array-level uniformity and reproducibility.
The uniformity and reproducibility of the photomemristor array, which are crucial for practical neuromorphic applications, are not sufficiently demonstrated. Although Figure S12 shows the photoresponse of eight devices under three illumination conditions, this dataset is limited for a 4×4 (16-device) array. Additional statistical validation is recommended, including dynamic response measurements from more array units under the same illumination conditions.

Reviewer #2

(Remarks to the Author)
This manuscript reports a TiO_2/PEDOT photomemristor that enables autonomous visual adaptation under mixed-light

conditions through a bioinspired dynamic water adsorption/desorption mechanism. The study is well organized, scientifically rigorous, and addresses a critical challenge in neuromorphic vision systems. The findings are timely and important, and are expected to advance the development of adaptive visual systems while attracting broad interest in this field. However, the following issues should be addressed to further clarify the underlying mechanism of the adaptive behavior and strengthen the manuscript's scientific rigor:

1. In Fig. 2c, the GIXRD pattern used to confirm the anatase phase of TiO_2 exhibits a high background and a low signal-to-noise ratio for the characteristic peaks, which weakens the reliability of phase identification. It is recommended that the authors remeasure the XRD pattern using a longer step integration time to improve the peak signal-to-noise ratio, or provide complementary characterizations such as Raman spectroscopy or HRTEM/SAED to independently verify the anatase crystal structure.
2. The band alignment of the TiO_2/PEDOT interface shown in Fig. S3 lacks experimental (e.g., UPS, XPS) or theoretical support, despite being central to the proposed carrier separation and transport mechanism. Supplementary data are needed to validate the band structure and strengthen the mechanistic interpretation.
3. Given that PEDOT is intrinsically sensitive to humidity, the device performance may fluctuate under practical environmental conditions. To evaluate environmental robustness and practical applicability, stability tests under varying humidity levels should be provided to quantify the influence of humidity on device performance.
4. The current uniformity characterization in Fig. S12 includes only 8 units from the 4 × 4 photomemristor array, which is insufficient to represent the performance consistency of the entire array. It is recommended that the authors provide the complete test data for all 16 units.
5. In Fig. S13, only five simple letter patterns are used for training, achieving 100% accuracy after merely three iterations. This suggests that the training dataset is overly simplistic and insufficient to rigorously validate the system's robustness and generalization capability in letter recognition tasks. Expanding the dataset with more diverse letter patterns is recommended to avoid potential overfitting and to strengthen the credibility of the recognition results.
6. The accuracy data in Fig. S15B exhibits significant fluctuations, which undermines the credibility of the ANN's recognition performance under the corresponding illumination condition. It is recommended that the ANN be retrained with optimized parameters (e.g., adjusted learning rate, increased training epochs) to improve training stability and result reliability.

Version 1:

Reviewer comments:

Reviewer #1

(Remarks to the Author)
The authors have well addressed my concerns. It can be accepted now.

Reviewer #2

(Remarks to the Author)
The authors have addressed my concerns and I suggest accepting this manuscript.

Open Access This Peer Review File is licensed under a Creative Commons Attribution 4.0 International License, which permits use, sharing, adaptation, distribution and reproduction in any medium or format, as long as you give appropriate credit to the original author(s) and the source, provide a link to the Creative Commons license, and indicate if changes were made.
In cases where reviewers are anonymous, credit should be given to 'Anonymous Referee' and the source.
The images or other third party material in this Peer Review File are included in the article's Creative Commons license, unless indicated otherwise in a credit line to the material. If material is not included in the article's Creative Commons license and your intended use is not permitted by statutory regulation or exceeds the permitted use, you will need to obtain permission directly from the copyright holder.
To view a copy of this license, visit https://creativecommons.org/licenses/by/4.0/

Professor Huanyu "Larry" Cheng, Ph. D.
James L. Henderson, Jr. Memorial Professor in Engineering
Associate Professor of Engineering Science and Mechanics
The Pennsylvania State University
212 Earth and Engineering Sciences Building
University Park, PA 16802-6812

(814) 863-5945
Fax: (814) 865-9974
huanyu.cheng@psu.edu
http://sites.psu.edu/chenggroup/

Reviewer #1:

This manuscript reports a TiO_2/PEDOT:PSS-based photomemristor capable of adaptive visual processing under mixed-light conditions, enabled by a light-modulated water adsorption/desorption process in the PEDOT:PSS layer. The device demonstrates pronounced synaptic current suppression under strong illumination—reaching levels even below the dark current—and, when implemented in a 4×4 array coupled with an artificial neural network, achieves a recognition accuracy of 91.3% under mixed-light scenarios. The study presents a promising bioinspired strategy for adaptive neuromorphic vision. While the results are compelling, several aspects of the experimental validation and mechanistic interpretation require clarification or additional support. Some revisions are therefore recommended to improve the rigor and completeness of the work. The following points should be addressed:

Our response: We thank the reviewer for the highly positive evaluation of our work. We also appreciate the insightful comments that help significantly improve the overall quality of this work. The manuscript has been thoroughly revised according to the reviewer's comments. The corresponding changes are highlighted in yellow in the revised manuscript.

1. Quantitative benchmarking of bioinspired vision adaptation. The analogy to human visual adaptation is currently discussed in a largely qualitative manner. To strengthen this claim, a quantitative comparison is recommended. Key adaptive metrics (e.g., dynamic range, adaptation time constants, suppression ratios) should be benchmarked against reported values for human photopic and scotopic vision, preferably summarized in a table or comparative figure.

Our Response: We thank the reviewer for this constructive suggestion. We agree that benchmarking against the human visual system can highlight the importance and novelty of the TiO_2/PEDOT:PSS-based photomemristor reported in this work. The main figures of merit for adaptation include adaptation time, working range, and power consumption. The comparison in the performance parameters shows that the TiO_2/PEDOT:PSS-based photomemristor exhibits significantly shorter adaptation times in both dark and bright environments than the human visual system, while maintaining comparably low power consumption. In addition, the TiO_2/PEDOT:PSS-based photomemristor also features a simple device structure and is compatible with scalable fabrication, which is advantageous for practical applications.

Our modification to the manuscript: We have included a comprehensive comparison of the adaptation performance, device structure, and operating mechanism of the TiO_2/PEDOT:PSS-based photomemristor with those of the human visual system in the revised manuscript.

"…As a result, the TiO_2/PEDOT:PSS-based photomemristor exhibits record-breaking suppression of photocurrent under bright illumination and demonstrates robust photopic–scotopic adaptation, significantly improving recognition accuracy under complex lighting conditions. To quantitatively and comprehensively highlight the bioinspired design advantages and performance of the TiO_2/PEDOT:PSS photomemristor, a detailed comparison between the as-fabricated device and the human visual system, in terms of structural and functional features, is provided in **Table S1**. The results show that the TiO_2/PEDOT:PSS-based photomemristor exhibits significantly shorter adaptation times in both dark and bright environments than the human visual system, while maintaining comparably low power consumption. In addition, the TiO_2/PEDOT:PSS-based photomemristor also features a simple device structure and is compatible with scalable fabrication, which is advantageous for practical applications."

Table S1. Comparison between the TiO_2/PEDOT:PSS-based photomemristor and the human visual system.

Parameter	This Work	Human vision system
Light adaptation	1.5 s	~5 min
Dark adaptation	<1 s	~20-30 min
Power consumption	~3.3 mW (max)	~3 mW (retina)
Adaptation mechanism	Adsorption/desorption of H_2O molecules	Rhodopsin bleaching–regeneration cycle
Structure	2 functional layers	~10 retinal layers
Thickness	~120 nm	~250 μm (retina)

2. Experimental validation of the oxygen-vacancy-based switching mechanism. The attribution of memristive switching to oxygen vacancy migration, as discussed in Figure 2g, lacks direct experimental evidence. To substantiate this mechanism, it is recommended to examine the dependence of device current on electrode area. If bulk oxygen vacancy migration dominates conduction, the current should scale proportionally with electrode area. Area-dependent electrical measurements would provide critical support for this interpretation.

Our Response: We agree that the oxygen-vacancy-based neuromorphic behavior of the TiO_2/PEDOT:PSS photomemristor has not been fully validated in the previous version. Oxygen-vacancy migration and filamentary conduction are two primary mechanisms underlying memristive switching (*doi: /10.1038/s41928-018-0092-2*). In bulk oxygen-vacancy-dominated conduction, the current scales with electrode area, whereas filamentary conduction exhibits weak area dependence due to localized conductive filaments. Notably, conventional filamentary memristors often suffer from inherent instability and poor uniformity. The random formation, rupture, and positional evolution of localized filaments typically result in abrupt switching behavior, large device-to-device variability, and limited cycling endurance—critical drawbacks that hinder their practical neuromorphic applications. In contrast, anatase TiO_2 has been extensively investigated for neuromorphic devices due to its intrinsic

defect chemistry and stable resistive switching behavior (*doi: 10.1039/D4TC00227J*). Specifically, oxygen vacancies in anatase TiO_2 can migrate under an external electric field, modulating local conductivity and forming uniform, tunable conductive paths. This process enables gradual conductance changes that closely resemble synaptic weight modulation in biological neural systems.

To further validate the oxygen-vacancy-based switching mechanism in our device, we have performed additional area-dependent electrical measurements on devices with different electrode sizes. As shown in the newly added Fig. S4, the current of the TiO_2/PEDOT:PSS photomemristor increases approximately linearly with electrode area, indicating that conduction scales proportionally with device area. This result strongly suggests that conduction is dominated by a bulk transport process rather than by localized filamentary conduction, consistent with the proposed oxygen-vacancy migration mechanism in the TiO_2/PEDOT:PSS photomemristor, which also contributes to its extraordinary stability and low sample-to-sample variations.

Our modification to the manuscript: We have included the size-dependent conductance of the TiO_2/PEDOT:PSS-based photomemristor in the revised manuscript.

"...These memristive characteristics can be explained by carrier migration and trapping at the TiO_2/PEDOT:PSS interface. This gradual and controllable conductance modulation is a key characteristic of oxygen-vacancy migration-based memristors, distinguishing our device from filamentary memristors and contributing to low device-to-device variability and extraordinary cycling endurance (*31*). To further validate the oxygen-vacancy-based switching mechanism in our device, additional area-dependent electrical measurements are performed on devices with different electrode sizes. As shown in **Fig. S4**, the current of the TiO_2/PEDOT:PSS photomemristor increases with electrode area, indicating that conduction scales proportionally with device area. This result strongly suggests that conduction is dominated by a bulk transport process rather than by localized filamentary conduction, consistent with the proposed oxygen-vacancy migration mechanism in the TiO_2/PEDOT:PSS photomemristor."

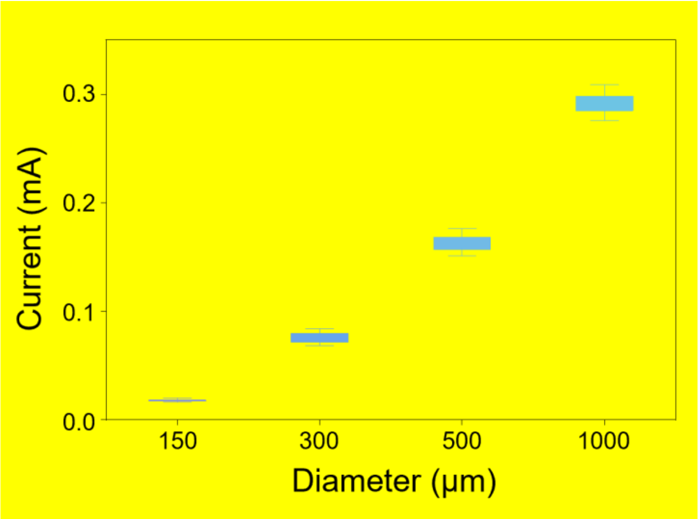

Fig. S4. Size-dependence of the current in the TiO_2/PEDOT:PSS-based photomemristor. Current of the TiO_2/PEDOT:PSS-based photomemristor as a function of units' size at a bias voltage of 2 V. Data are presented as mean ± SD (n = 5 independent measurements).

3. Quantification of photothermal effects under illumination. The light-adaptive behavior is attributed to photothermal heating-induced water desorption in the PEDOT:PSS layer. However, the manuscript does not establish a direct quantitative relationship between illumination intensity and the resulting temperature rise at the device surface. Experimental measurements—such as infrared thermography—to map surface temperature distributions under different light intensities are strongly recommended.

Our Response: We thank the reviewer for this valuable suggestion. We agree that photothermal heating has not been directly confirmed experimentally. To quantitatively evaluate the photothermal effect under illumination, we have performed systematic infrared (IR) thermography measurements at four illumination intensities (0, 100, 200, and 320 mW cm^{-2}) at a constant room temperature of 20 °C. The results show that the temperature of the TiO_2/PEDOT:PSS photomemristor increases monotonically with illumination duration. For example, under 320 mW cm^{-2} illumination for 7.5 s, the device temperature reaches 32.7 °C. Furthermore, the ultimate temperature of the photomemristor also rises with increasing

illumination intensities. Collectively, these results confirm the presence of a pronounced photothermal effect in the TiO_2/PEDOT:PSS photomemristor, which can be attributed to the unique light absorption properties of PEDOT:PSS and TiO_2. This photothermal effect facilitates water desorption from the PEDOT:PSS film, thereby modulating the conductance of the device and contributing to the observed light-adaptive behavior.

Our modification to the manuscript: We have included IR thermography images of the TiO_2/PEDOT:PSS-based photomemristor under different illumination intensities and durations in the revised manuscript.

"...This process reduces the hydronium ion (H_3O^+) concentration and consequently leads to the decreased conductivity of PEDOT:PSS, which suppresses the synaptic current of the TiO_2/PEDOT:PSS-based photomemrsistor in strong light (**Fig. 4a**). Systematic infrared (IR) thermography measurements at four illumination intensities (0, 100, 200, and 320 mW cm^{-2}) at a constant room temperature of 20 °C quantitatively evaluate the photothermal effect under illumination (**Movie S1** and **Fig. S11**). The results show that the temperature of the TiO_2/PEDOT:PSS photomemristor increases monotonically with illumination intensities. For example, after 7.5 s of illumination, the temperature of the photomemristor increases from 24.2 to 32.7℃ as the illumination intensity rises from 100 to 320 mW cm^{-2}. Furthermore, its ultimate temperature also rises with increasing illumination duration. Collectively, these results confirm the presence of a pronounced photothermal effect in the TiO_2/PEDOT:PSS photomemristor, which can be attributed to the unique light absorption properties of PEDOT:PSS and TiO_2."

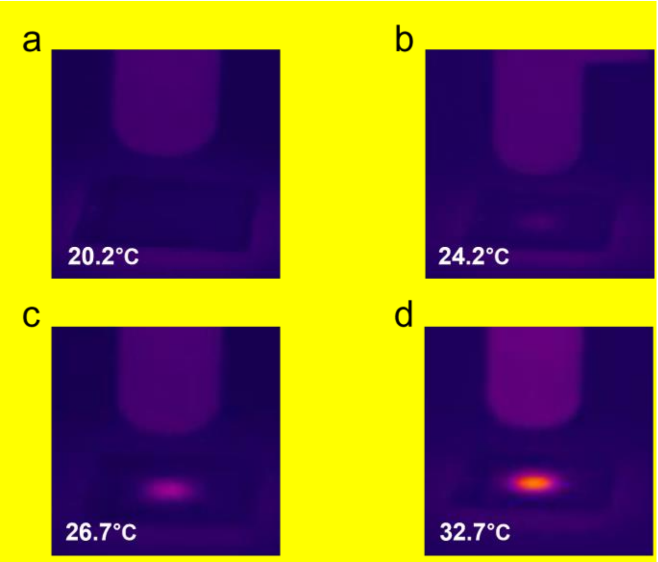

Fig. S11. Photothermal effect in the TiO_2/PEDOT:PSS-based photomemristor. Infrared (IR) thermal images of the TiO_2/PEDOT:PSS-based photomemristor **(a)** in the dark and **(b-d)** under 7.5 s of UV illumination with intensities of **(b)** 100, **(c)** 200, and **(c)** 320 mW cm^{-2} (365 nm), respectively.

4. Revision of resistance extraction methodology in Figure S8b. The resistance analysis presented in Figure S8b is methodologically inappropriate. Averaging V/I values across different points on nonlinear I–V curves does not yield a physically meaningful resistance. Instead, resistance should be consistently extracted from each I–V trace, for example by calculating the inverse slope of the linear (ohmic) region. The resulting resistance values should then be statistically compared across temperatures and device samples.

Our Response: We thank the reviewer for this valuable suggestion. Photomemristors often exhibit nonlinear *I–V* curves because current transport is governed by field-dependent conduction and interface barriers rather than simple Ohmic behavior. The nonlinear characteristics observed in our devices arise from these mechanisms and interface effects. We agree that it is inappropriate to average resistance values calculated across different applied voltages. Therefore, the resistance values reported in this work

have been updated by extracting specifically from the linear regime of the *I–V* curves and averaging over eight independent samples to ensure statistical reliability. Notably, the resistance of the PEDOT:PSS film gradually increases with temperature, consistent with the behavior of the TiO_2/PEDOT:PSS photomemristor. These results confirm that the adaptive behavior of the TiO_2/PEDOT:PSS photomemristor originates from the tunable resistance of PEDOT:PSS, modulated by water absorption and desorption.

Our modification to the manuscript: We have updated the resistance values of the PEDOT:PSS film measured from eight independent samples in the revised manuscript.

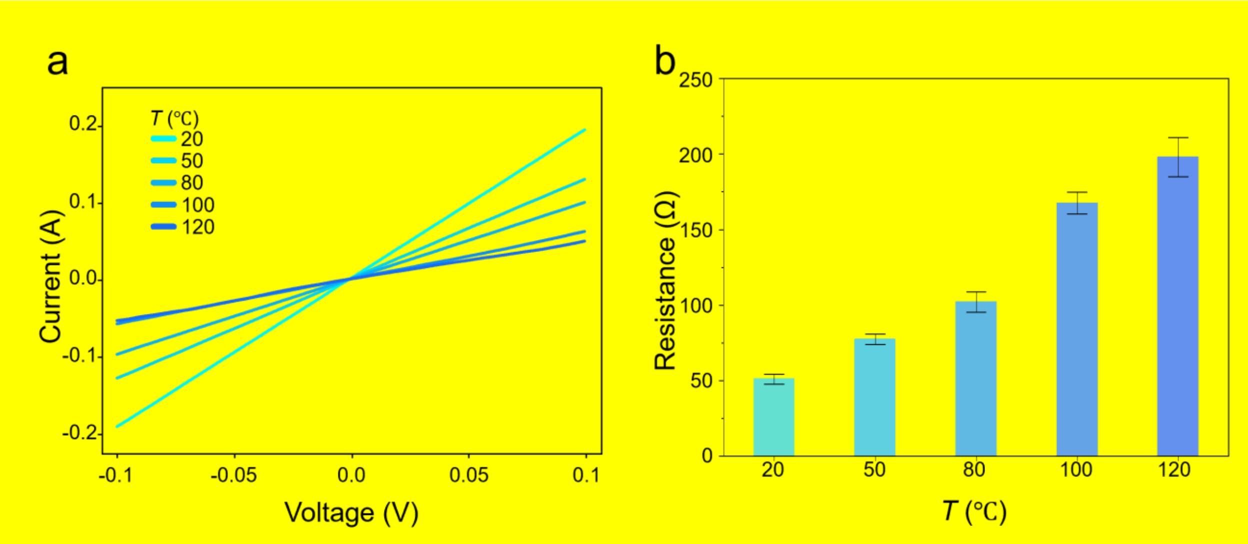

Fig. S12. Temperature-dependent electrical conductivity of PEDOT:PSS films. **(a)** *I-V* curves and **(b)** electrical resistance of PEDOT:PSS films extracted from the linear region of the *I-V* curves at different temperatures. Data are presented as mean ± SD (n = 8 independent samples).

5. Array-level uniformity and reproducibility. The uniformity and reproducibility of the photomemristor array, which are crucial for practical neuromorphic applications, are not sufficiently demonstrated. Although Figure S12 shows the photoresponse of eight devices under three illumination conditions, this dataset is limited for a 4×4 (16-device) array. Additional statistical validation is recommended, including dynamic response measurements from more array units under the same illumination conditions.

Our Response: We thank the reviewer for this valuable suggestion. To further demonstrate the uniformity and reproducibility of the photomemristor array, we have measured the photoresponse of all 16 devices in the 4×4 array under different illumination conditions (0, 200, and 320 mW cm^{-2}). The results show that the spike current response of each device is consistent across illumination conditions. All units exhibit remarkably suppressed postsynaptic current and light adaptation, with the same inflection point under bright illumination (320 mW cm^{-2}). Statistical analysis of the spike current responses further confirms excellent device-to-device uniformity and reproducibility. Collectively, these results demonstrate that the TiO_2/PEDOT:PSS photomemristor array is well suited for practical neuromorphic applications.

Our modification to the manuscript: We have included the photoresponse of all units in the 4×4 array under different illumination conditions in the revised manuscript.

"...An artificial neural network (ANN) is incorporated into the highly adaptive 4 × 4 photomemristor array to form the artificial visual system (**Fig. 5c**). To examine the uniformity of the photomemristor array, the PSC of sixteen units under a 1.5 V pulse voltage in three illumination conditions—dim (0 mW cm^{-2}, **Fig. S17a**), moderate (200 mW cm^{-2}, **Fig. S17b**), and bright (320 mW cm^{-2}, **Fig. S17c**) light is measured and compared."

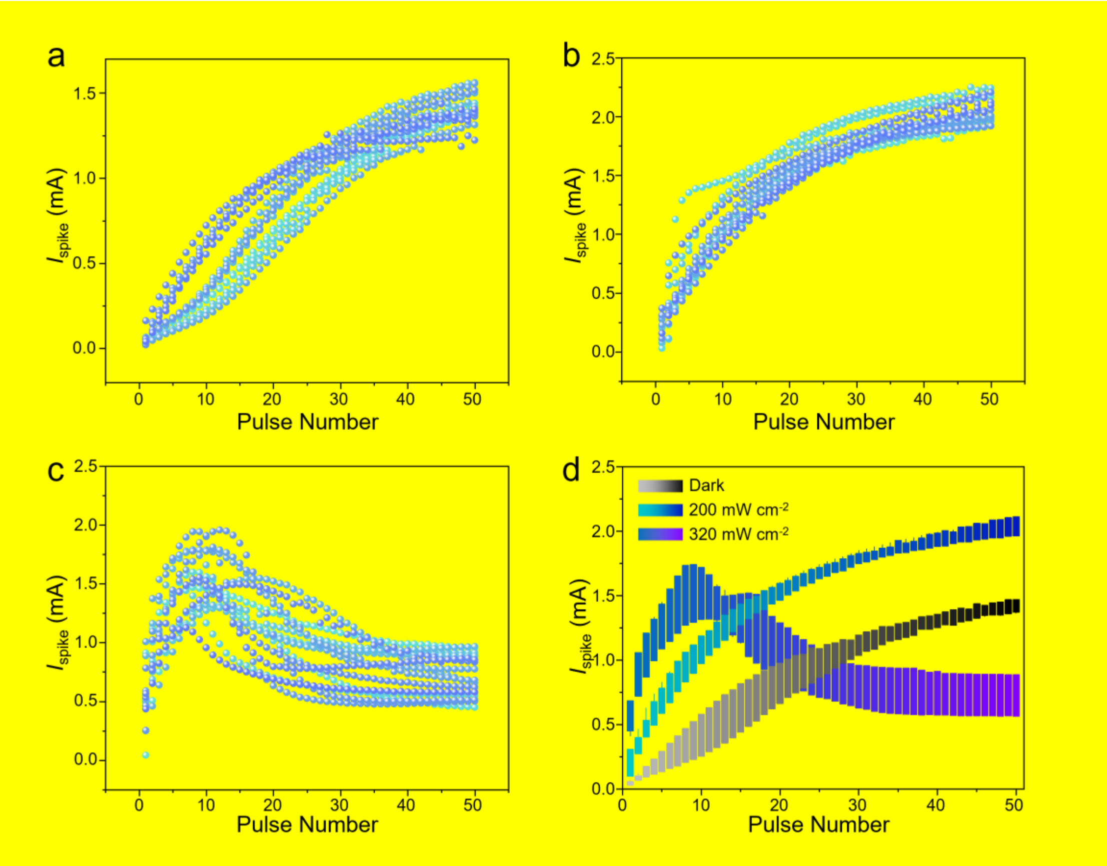

Fig. S17. Sample-to-sample variations in TiO_2/PEDOT:PSS-based photomemristors. I_{spike} of 16 units in a 4 × 4 TiO_2/PEDOT:PSS-based photomemristor array as a function of the pulse number under different light intensities: **(a)** 0 mW cm^{-2}, **(b)** 200 mW cm^{-2}, and **(c)** 320 mW cm^{-2}. **(d)** Statistics of I_{spike} under different light intensities. Data are presented as mean ± SD (n = 16 independent samples). V_{bias} = 1.5 V, P_w = 100 ms, Δt = 50 ms.

PennState

Professor Huanyu “Larry” Cheng, Ph. D.
James L. Henderson, Jr. Memorial Professor in Engineering
Associate Professor of Engineering Science and Mechanics
The Pennsylvania State University
212 Earth and Engineering Sciences Building
University Park, PA 16802-6812

(814) 863-5945
Fax: (814) 865-9974
huanyu.cheng@psu.edu
http://sites.psu.edu/chenggroup/

Reviewer #2:

This manuscript reports a TiO_2/PEDOT photomemristor that enables autonomous visual adaptation under mixed-light conditions through a bioinspired dynamic water adsorption/desorption mechanism. The study is well organized, scientifically rigorous, and addresses a critical challenge in neuromorphic vision systems. The findings are timely and important, and are expected to advance the development of adaptive visual systems while attracting broad interest in this field. However, the following issues should be addressed to further clarify the underlying mechanism of the adaptive behavior and strengthen the manuscript's scientific rigor:

Our Response: We thank the reviewer for the highly positive and insightful evaluation of our work, as well as the recognition of the scientific significance and application value of our TiO_2/PEDOT photomemristor with bioinspired autonomous visual adaptation capability. We greatly appreciate the reviewer's affirmation of the study's organization, scientific rigor and the timeliness of our findings for the development of neuromorphic vision systems. We have carefully addressed all the issues raised by the reviewer to further clarify the underlying mechanism of the adaptive behavior and enhance the scientific rigor of the manuscript. Corresponding revisions, supplementary experimental characterizations and in-depth mechanistic analyses have been completed, with all modified content highlighted in yellow in the revised manuscript. We believe these revisions have significantly improved the completeness and persuasiveness of our work.

1. In Fig. 2c, the GIXRD pattern used to confirm the anatase phase of TiO_2 exhibits a high background and a low signal-to-noise ratio for the characteristic peaks, which weakens the reliability of phase

identification. It is recommended that the authors remeasure the XRD pattern using a longer step integration time to improve the peak signal-to-noise ratio, or provide complementary characterizations such as Raman spectroscopy or HRTEM/SAED to independently verify the anatase crystal structure.

Our Response: We thank the reviewer for the careful evaluation and constructive suggestion. We agree that the small thickness of the TiO_2 film leads to a low signal-to-noise ratio in the GIXRD pattern, which limits clear identification of the anatase phase. To address this issue, Raman spectroscopy has been performed, revealing distinct characteristic peaks of anatase TiO_2 (e.g., 143 cm^{-1}). Collectively, the revised GIXRD and Raman results confirm the formation of high-quality anatase TiO_2 thin films.

Our modification to the manuscript: We have conducted Raman spectroscopy and included these results in the revised manuscript.

"...Grazing-incidence X-ray diffraction (GIXRD) confirms that the TiO_2 layer adopts the anatase phase (**Fig. 2c**), which can be attributed to the relatively low annealing temperature. Raman spectroscopy further confirms the anatase crystal structure of the TiO_2 film, with characteristic peaks located at 143, 399, 492, and 633 cm^{-1} (**Fig. S2**), corresponding well to the typical vibrational modes of anatase TiO_2 (Eg, B_{1g}, A_{1g}, and E_g, respectively)."

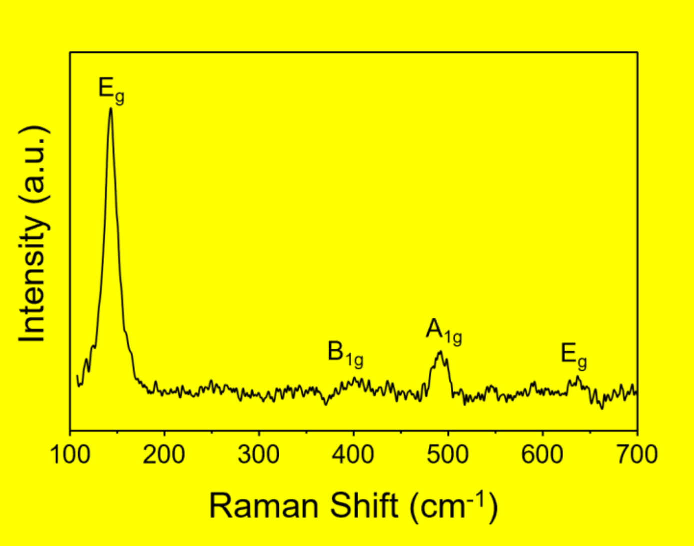

Fig. S2. Characterization of crystal structures of TiO_2 thin film. Raman spectrum of the TiO_2 film. The characteristic peaks at approximately 143 cm^{-1}, 399 cm^{-1}, 492 cm^{-1}, and 633 cm^{-1} are attributed to the E_g, B_{1g}, A_{1g}, and E_g vibrational modes of anatase TiO_2, respectively.

2. The band alignment of the TiO_2/PEDOT interface shown in Fig. S3 lacks experimental (e.g., UPS, XPS) or theoretical support, despite being central to the proposed carrier separation and transport mechanism. Supplementary data are needed to validate the band structure and strengthen the mechanistic interpretation.

Our Response: We thank the reviewer for this constructive suggestion. We agree that experimental measurements of the band structures of TiO_2 and PEDOT:PSS are essential for determining the band alignment of the TiO_2/PEDOT:PSS heterojunction and validating the proposed operating mechanism. Accordingly, ultraviolet photoelectron spectroscopy (UPS) and ultraviolet–visible (UV–vis) absorption spectroscopy have been performed to characterize the band structures of TiO_2 and PEDOT:PSS. The UPS results reveal the conduction band (CB) of TiO_2 (~3.74 eV) and the highest occupied molecular orbital (HOMO) of PEDOT:PSS (~3.16 eV) relative to the vacuum level. The band gaps of TiO_2 and PEDOT:PSS are determined as 3.32 and 1.52 eV from UV–vis absorption spectra using Tauc plot analysis, assuming an indirect transition for TiO_2 and a direct transition for PEDOT:PSS. Based on these experimentally derived parameters, the energy band alignment of the TiO_2/PEDOT:PSS heterojunction is further constructed.

Our modification to the manuscript: We have added the detailed experimental results of UPS and UV-Vis absorption spectroscopy of TiO_2 and PEDOT:PSS in the revised manuscript.

“…This result strongly suggests that conduction is dominated by a bulk transport process rather than by localized filamentary conduction, consistent with the proposed oxygen-vacancy migration mechanism in the TiO_2/PEDOT:PSS photomemristor. The ultraviolet photoelectron spectroscopy (UPS) results reveal the conduction band (C_B) of TiO_2 (~3.74 eV) and the highest occupied molecular orbital (HOMO) of PEDOT:PSS (~3.16 eV) relative to the vacuum level (**Fig. S5ac**). The band gaps of TiO_2 and PEDOT:PSS are determined as 3.32 and 1.52 eV from UV–vis absorption spectra using Tauc plot analysis, assuming an indirect transition for TiO_2 and a direct transition for PEDOT:PSS (**Fig. S5bd**). Based on these experimentally derived parameters, the energy band alignment of the TiO_2/PEDOT:PSS heterojunction is constructed (**Fig. S6**), and the built-in electric field arising from carrier diffusion across the interface is schematically illustrated in **Fig. 2g**. Under positive bias, electrons are injected from the FTO electrode and trapped by oxygen vacancies of TiO_2, which weakens the built-in electric field in the depletion region…”

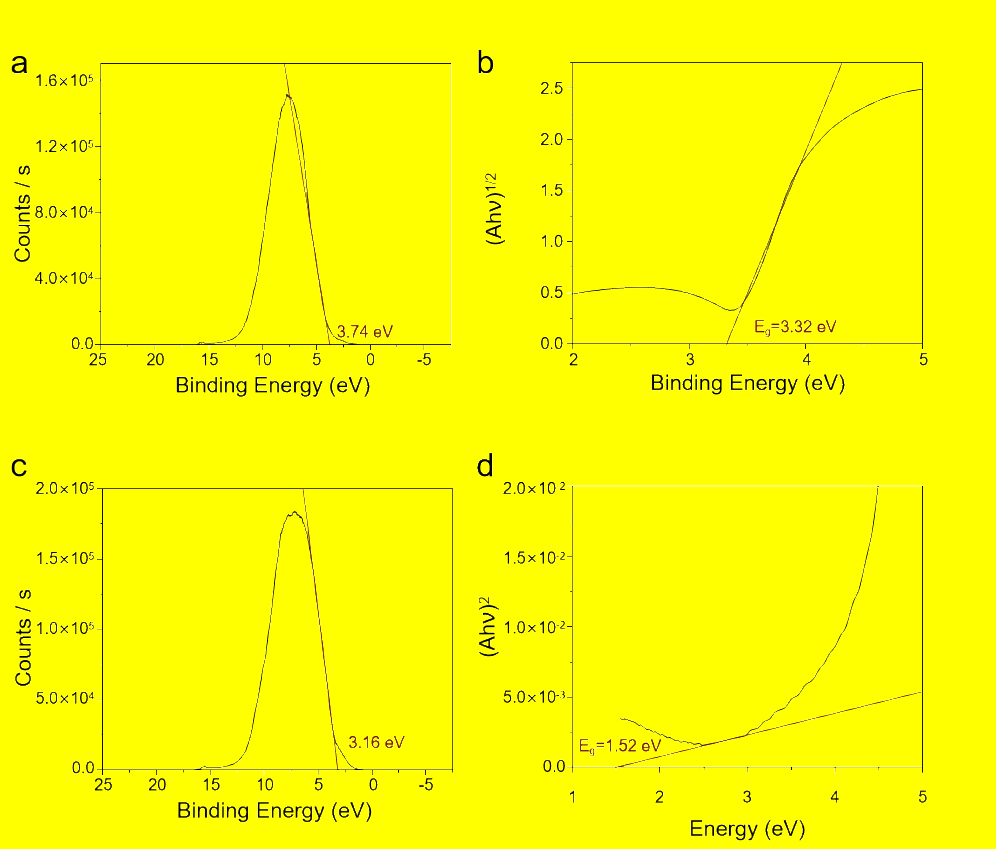

Fig. S5. Band structure characterization of TiO_2 and PEDOT:PSS. (**a**) Conduction band (C_B) level of TiO_2 determined by ultraviolet photoelectron spectroscopy (UPS). (**b**) Tauc plot derived from the UV–vis absorption spectrum of TiO_2 for estimating the indirect bandgap. (**c**) The lowest unoccupied molecular orbital (LUMO) level of PEDOT:PSS determined by UPS. (**d**) Tauc plot derived from the UV–vis absorption spectrum of PEDOT:PSS for estimating the direct bandgap.

We have also updated the band alignment diagram of the TiO_2/PEDOT:PSS heterojunction in the revised manuscript.

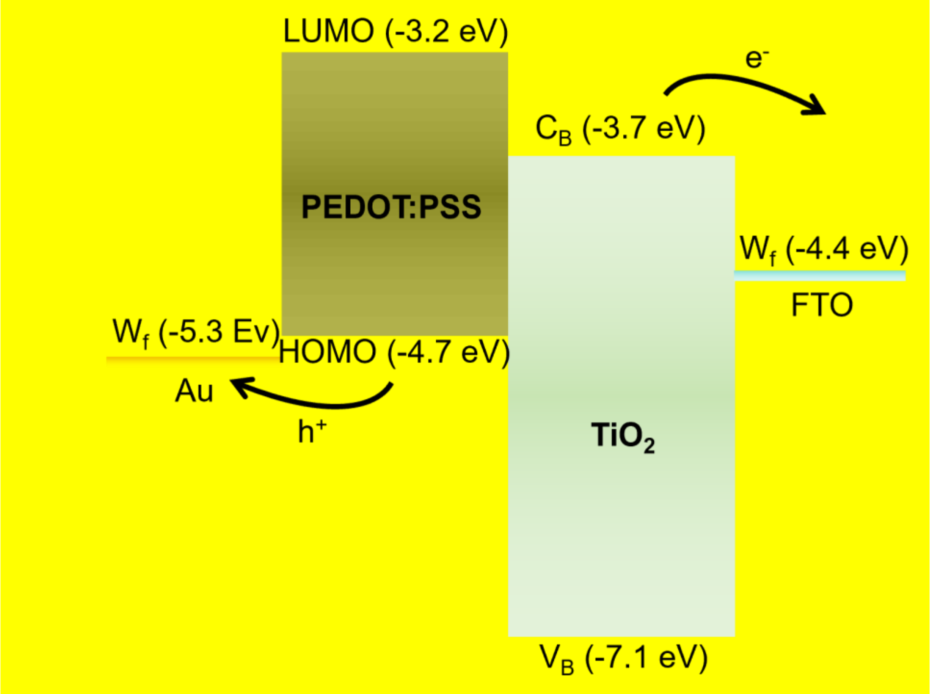

Fig. S6. Energy band alignment of the photomemristor. Schematic illustrating the energy band alignment of the TiO_2/PEDOT:PSS photomemristor derived from the measured band structure of TiO_2 and PEDOT:PSS.

3. Given that PEDOT is intrinsically sensitive to humidity, the device performance may fluctuate under practical environmental conditions. To evaluate environmental robustness and practical applicability,

stability tests under varying humidity levels should be provided to quantify the influence of humidity on device performance.

Our Response: We thank the reviewer for this valuable suggestion. We agree that humidity may influence the adaptation performance of the TiO_2/PEDOT:PSS-based photomemristor by interfering with the water adsorption/desorption process. To evaluate the environmental robustness of the device, we have performed additional stability measurements under different relative humidity (RH) conditions ranging from 30% to 92%. The results show that the photomemristor maintains its light-adaptive behavior over a wide humidity range, although the adaptation time slightly increases from ~1 to 1.5 s with increasing humidity. This behavior can be attributed to the stronger water adsorption at higher RH levels, which requires a longer illumination time to induce sufficient photothermal heating for water desorption.

Our modification to the manuscript: We have included the photoelectric response of the TiO_2/PEDOT:PSS photomemristor under varying humidity levels in the revised manuscript.

"…However, immersing the TiO_2/PEDOT:PSS-based photomemristor in highly humid environments recovers its light adaptation (**Fig. 4e**). These results indicate that the light adaptation originates from the temperature-regulated water absorption rather than the temperature itself. Further reliability evaluation of the TiO_2/PEDOT:PSS-based photomemristor under varying environmental conditions measures its photoresponse at three relative humidity (RH) levels (30%, 54%, and 92%) under UV illumination (320 mW cm^{-2}) (**Fig. S12**). Notably, the adaptive knee point shifts from the 6th pulse (0.9 s) at 30% RH to the 11th pulse (1.65 s) at 92% RH, indicating a clear humidity-dependent delay. The adaptive behaviour occurs earlier in low-humidity environments due to the limited ambient water molecules, accelerating dehydration of the PEDOT:PSS film. In contrast, under high RH of 92%, adaptation appears slightly later because abundant water vapor slows water loss from the PEDOT:PSS layer. Nevertheless, within the preset operation window (7.5 s), substantial water desorption occurs under

illumination for all humidity conditions, resulting in nearly identical final current. These results confirm that the TiO_2/PEDOT:PSS photomemristor can operate reliably across a wide range of environmental humidity levels. The weak dependence on ambient humidity is attributed to the spontaneous adsorption of water molecules by the hygroscopic PEDOT:PSS film, which reaches near-saturation under normal RH."

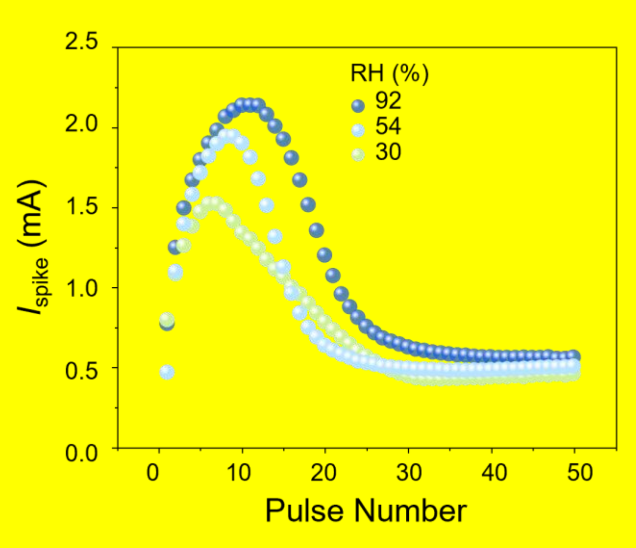

Fig. S13. Influence of humidity on the adaptation performance of the TiO_2/PEDOT:PSS-based photomemristor. I_{spike} of the TiO_2/PEDOT:PSS-based photomemristor under 320 mW cm^{-2} illumination at relative humidity (RH) levels of 92%, 54%, and 30%.

4. The current uniformity characterization in Fig. S12 includes only 8 units from the 4 × 4 photomemristor array, which is insufficient to represent the performance consistency of the entire array. It is recommended that the authors provide the complete test data for all 16 units.

Our Response: We thank the reviewer for this rigorous and constructive suggestion, which is critical for ensuring the comprehensiveness and representativeness of the current uniformity characterization of the 4×4 photomemristor array. We fully agree that testing only 8 units is insufficient to reflect the overall

performance consistency of the entire array, and have accordingly supplemented the complete test data for all 16 device units as recommended. To address this comment, we have re-conducted the current uniformity test for all 16 units of the 4×4 photomemristor array, and collected the current response data of each unit under the three light intensities (0, 200, 320 $mWcm^{-2}$). The complete test data of all units fully reflect the current uniformity and performance consistency of the entire photomemristor array, providing more rigorous and comprehensive experimental support for the array-level performance of our device.

Our modification to the manuscript: We have fully revised Fig. S12 by replacing the original partial data (8 units) with the complete dynamic photoresponse data of all 16 units of the 4×4 photomemristor array under the three tested illumination conditions, with each unit's response curve clearly distinguished for intuitive comparison.

"...An artificial neural network (ANN) is incorporated into the highly adaptive 4 × 4 photomemristor array to form the artificial visual system (**Fig. 5c**). To examine the uniformity of the photomemristor array, the PSC of sixteen units under a 1.5 V pulse voltage in three illumination conditions—dim (0 mW cm^{-2}, **Fig. S17a**), moderate (200 mW cm^{-2}, **Fig. S17b**), and bright (320 mW cm^{-2}, **Fig. S17c**) light is measured and compared."

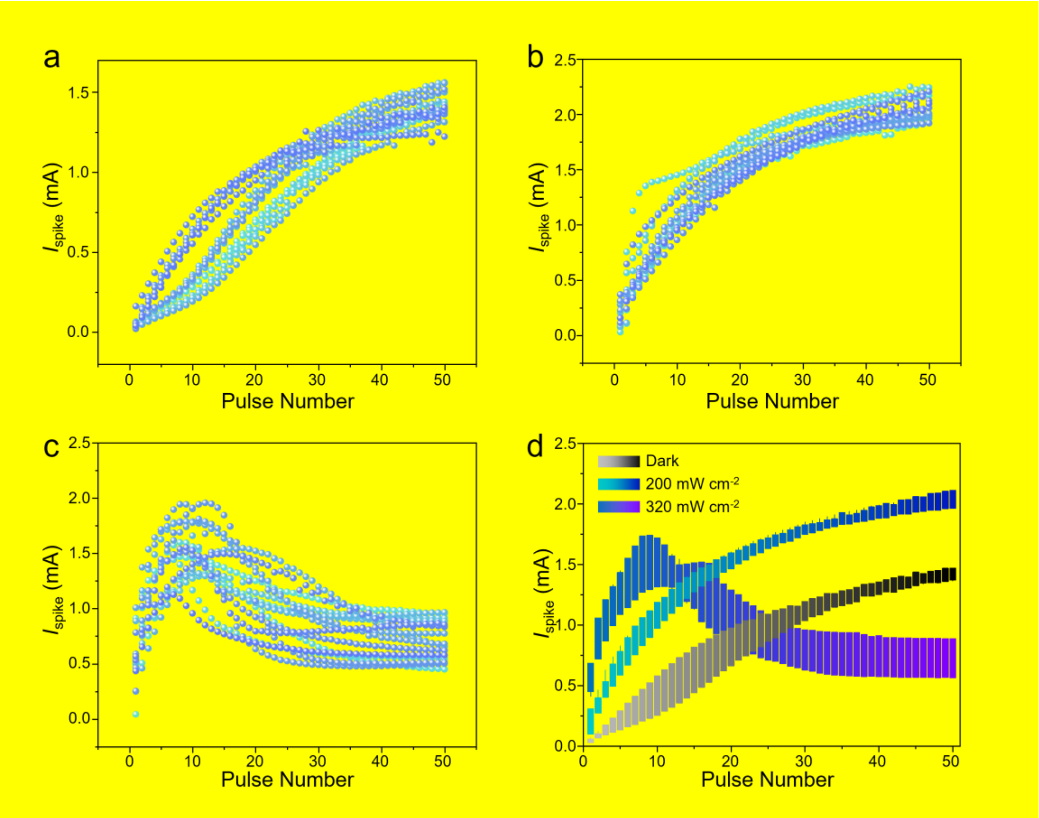

Fig. S17. Sample-to-sample variations in TiO_2/PEDOT:PSS-based photomemristors. I_{spike} of 16 units in a 4 × 4 TiO_2/PEDOT:PSS-based photomemristor array as a function of the pulse number under different light intensities: **(a)** 0 mW cm^{-2}, **(b)** 200 mW cm^{-2}, and **(c)** 320 mW cm^{-2}. **(d)** Statistics of I_{spike} under different light intensities. Data are presented as mean ± SD (n = 16 independent samples). V_{bias} = 1.5 V, P_w = 100 ms, Δt = 50 ms.

5. In Fig. S13, only five simple letter patterns are used for training, achieving 100% accuracy after merely three iterations. This suggests that the training dataset is overly simplistic and insufficient to rigorously validate the system's robustness and generalization capability in letter recognition tasks. Expanding the dataset with more diverse letter patterns is recommended to avoid potential overfitting and to strengthen the credibility of the recognition results.

Our Response: We thank the reviewer for this constructive suggestion. We agree that a more diverse dataset can better demonstrate the robustness and generalization capability of the photomemristor-based visual system. We have expanded the training dataset by incorporating additional letter patterns (K, D, O, Z, L). The results show that the system can successfully recognize a larger set of patterns with consistently high accuracy after training. These results further confirm the robustness and reliability of the proposed system.

Our modification to the manuscript: We have fully revised Fig. 18 by replacing the original 5-letter patterns recognition task with the 10-letter patterns recognition task.

"...The good linearity of spike-timing-dependent plasticity in the TiO_2/PEDOT:PSS photomemristor is beneficial for improving prediction accuracy. The performance of the artificial visual system is evaluated with ten standard letter patterns, showcasing a high accuracy of over 90% after only 7 iterations (**Fig. S18**)."

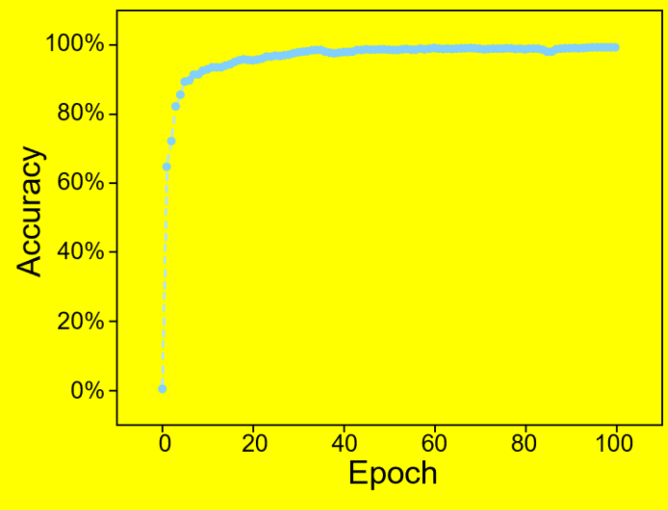

Fig. S18 Artificial neural network (ANN) prediction accuracy versus training epochs. Prediction accuracy of the ANN fed with the images of ten standard letter patterns (A, C, F, I, U, K, D, O, Z, L) as a function of epochs.

6. The accuracy data in Fig. S15B exhibits significant fluctuations, which undermines the credibility of the ANN's recognition performance under the corresponding illumination condition. It is recommended that the ANN be retrained with optimized parameters (e.g., adjusted learning rate, increased training epochs) to improve training stability and result reliability.

Our Response: We thank the reviewer for this constructive suggestion. We agree that fluctuations in the accuracy data may affect the reliability of the recognition results. When the dataset is relatively small, an excessively large hidden layer may lead to issues such as overfitting, unstable gradients, and difficulties in optimization. Following the reviewer's recommendation, we increased the training dataset from 5 to 10 letter patterns and reduced the number of hidden layers from 100 to 10 to reduce fluctuations in the recognition results. The updated results show significantly improved training stability, with much smoother accuracy evolution and consistent convergence behavior. These improvements enhance the reliability of the recognition performance under the corresponding illumination condition.

Our modification to the manuscript: We have updated the neural network framework diagram (Fig. 5c) and the corresponding training results (Figs. 5f and S20) in the revised manuscript.

"...For example, the recognition accuracy of the ANN fed by the PSC mapping at 0.15 s remains low (~ 10%) regardless of interactions. With the time increased to 7.5 s to trigger the light adaptation, the recognition accuracy increases significantly to 93.7% after 100 iterations (**Fig. 5f**). Besides the mixed-light environment, the artificial visual system also allows for image recognition in monochromatic environments, including bright objects in moderate-light conditions (**Fig. S19a**), and bright (**Fig. S19b**) and moderate-lighted (**Fig. S19c**) objects in dim conditions. Due to the remarkable PSC suppression, the "F" pattern shows a decreasing gray level, which becomes even lower than that corresponding to the dark background (**Fig. S19b**). Despite the overlap between the dark current and the suppressed PSC in

bright light, the ANN is capable of recognizing the image with high accuracy (>95%) by applying more pulses (**Fig. S20**). Furthermore, as the training iteration increases, the fluctuation in recognition accuracy is gradually reduced to result in a smoother evolution of the accuracy curve, which demonstrates the high reliability of the proposed neural network. Overall, the artificial visual system combined with the ANN can afford accurate image recognition in both monochromatic and mixed-light conditions with a process time of 7.5 s (**Fig. 5g**)."

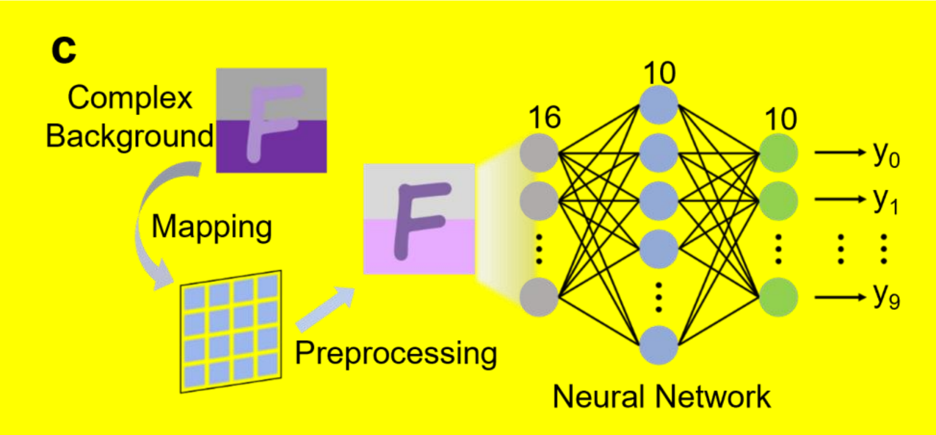

Fig. 5c Working flow of the artificial visual system for image recognition.

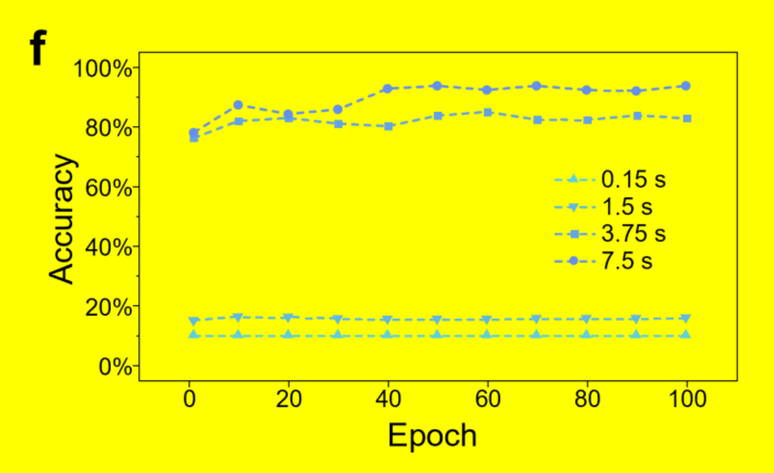

Fig. 5f Prediction accuracy of the artificial neural network (ANN) fed by the image detected by the photomemristor array as a function of epochs.

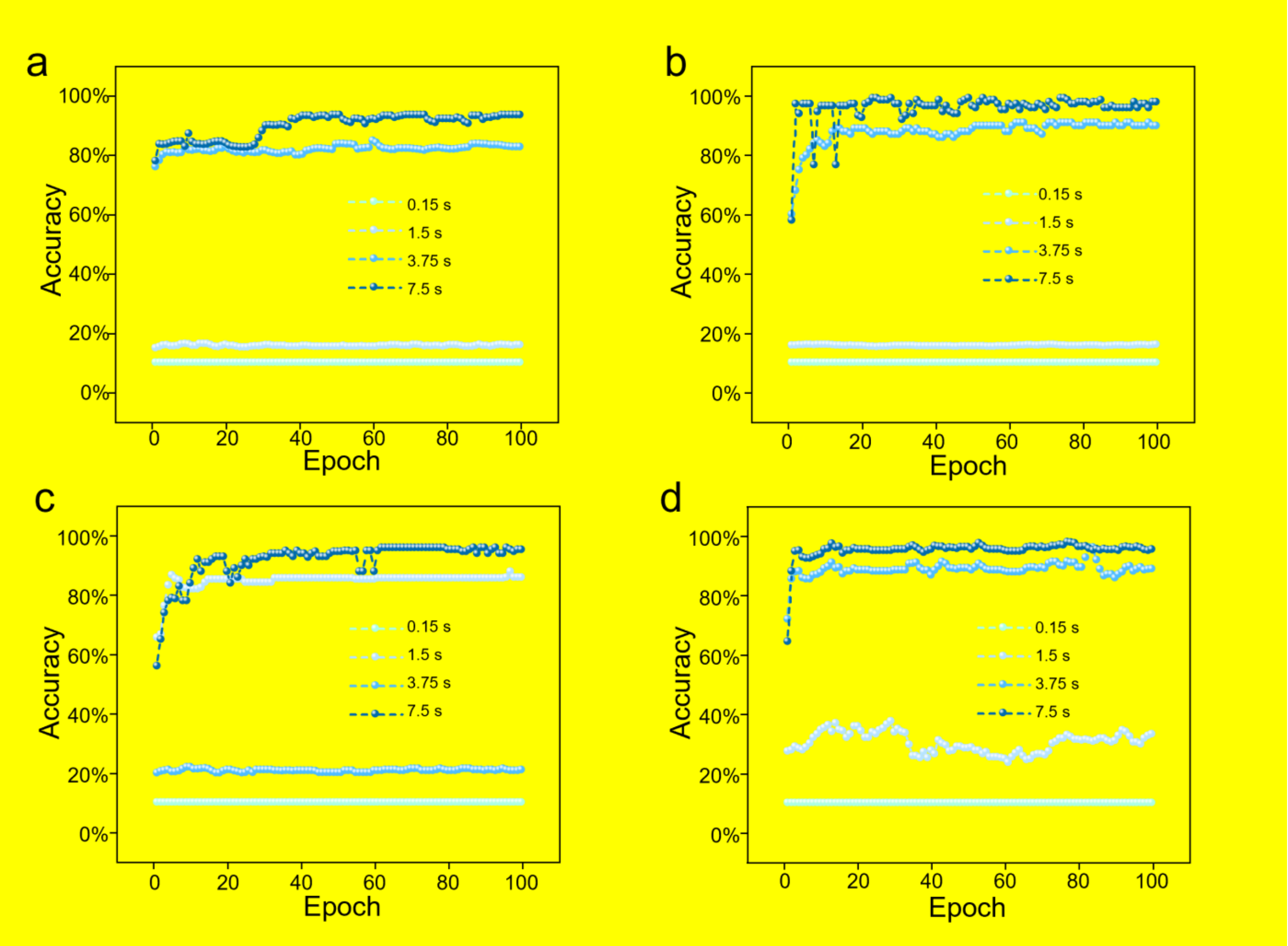

Fig. S20. ANN prediction accuracy under varying illumination conditions. Prediction accuracy of the ANN as a function of epochs in different illumination conditions, including **(a)** moderate-light “F” (200 mW cm^{-2}) against a mixed-light background (0 and 320 mW cm^{-2} for the top and bottom panel), **(b)** bright “F” (320 mW cm^{-2}) against a moderate-light background (200 mW cm^{-2}), **(c)** bright “F” (320 mW cm^{-2}) against a dim background (0 mW cm^{-2}), and **(d)** moderate-light “F” (200 mW cm^{-2}) against a dim background (0 mW cm^{-2}). The postsynaptic current distribution (or images) detected by the array at different time lapses (i.e., 0.15, 1.5, 3.75, 7.5s) are fed to the ANN for training.
